# Quantitative Assessment and Verification of Quantum Neural Network Security Based on Violations of the Bell Inequality

**DOI:** 10.3390/e28080860

**Published:** 2026-08-01

**Authors:** Yulu Zhang

**Affiliations:** School of Materials Science and Engineering, Hainan University, Haikou 570228, China; ylz@hainanu.edu.cn

**Keywords:** quantum neural networks, Bell’s inequality, quantum entanglement, quantitative security assessment and verification

## Abstract

In response to the current research landscape, which lacks unified quantitative standards and a reproducible verification framework for assessing the security of quantum neural networks (QNNs), this paper proposes a quantitative evaluation system and verification scheme for QNN security based on the violation characteristics of CHSH-type Bell inequalities. This method treats quantum entanglement as the core element of intrinsic security and establishes a controlled-variable controlled experiment involving purely classical models, non-entangled QNNs, weakly entangled QNNs, and strongly entangled QNNs. Numerical simulations were conducted using the Iris dataset; the presence of quantum entanglement was determined using the CHSH statistic, and a quantitative metric system centered on the normalized CHSH observation metric Q was constructed. Based on the theoretical limits of Bell’s inequalities, a normalization derivation was performed to establish the theoretical constraint interval of 0≤Q≤1; the threshold values of 0.3 and 0.7 obtained from the simulations are applicable only to the experimental scenarios described in this paper and serve solely as a reference for grouping data within the experiment; they do not possess universal validity for determination. Simulation results show that the CHSH values for the weakly and strongly entangled QNN experimental groups can reach 2.8284, significantly exceeding the theoretical limits of classical locality. These values correspond to an observation metric of Q=0.9838 and a privacy protection strength of P=0.8854, with the security level rated as high. The CHSH values of the non-entangled QNN and the purely classical model do not exceed the classical threshold of 2; they exhibit no quantum nonlocality or quantum advantage, and their security level is rated as low. This paper advances the security evaluation of QNNs from qualitative, empirical judgments to verifiable quantitative classification, providing a theoretical basis and evaluation framework for the practical application of quantum neural networks in highly security-sensitive scenarios such as privacy-preserving computing.

## 1. Introduction

As a cutting-edge interdisciplinary field at the intersection of quantum physics and machine learning, quantum machine learning has broken through the inherent paradigms of traditional classical information processing. By leveraging fundamental properties of quantum mechanics, such as quantum superposition, quantum entanglement, and quantum coherence, it has established an entirely new intelligent data processing framework, demonstrating immense potential in areas such as complex task modeling, high-dimensional feature extraction, and nonlinear fitting. At the same time, emerging scenarios, such as high-precision quantum experimental analysis, intelligent processing of classified data, and high-security privacy-preserving computing, have created an urgent need for high-performance, highly reliable data processing technologies, driving quantum machine learning from theoretical exploration toward practical implementation. Therefore, systematically analyzing the physical mechanisms, performance advantages, and security vulnerabilities of quantum machine learning and clarifying the intrinsic relationship between quantum correlation properties and model security holds significant value for both theoretical research and engineering applications.

As security concerns regarding variational quantum neural networks have garnered increasing attention, numerous studies have explored attack paradigms, privacy protection, robustness evaluation, and theories of quantum advantage. In terms of modeling attacks on quantum machine learning, Papadopoulos et al. proposed a numerical gradient inversion attack targeting variational quantum circuits, demonstrating that an adversary can use gradient information to reconstruct training samples and reveal privacy leakage risks during the parameter optimization phase [1]. Addressing the threat of model reverse engineering, Ghosh and Ghosh researched quantum imitation games, verifying that a finite number of input–output queries can replicate the functionality of a quantum machine learning model, thereby highlighting the security risks posed by model theft [2]. Building on this, Fu et al. further examined QNN extraction attacks under noisy conditions, quantifying the impact of decoherence noise on model stealability; however, they did not distinguish between the differing security constraints imposed by exogenous noise perturbations and endogenous quantum correlations within the circuit [3]. Innan et al. systematically mapped out the threat vectors across the entire lifecycle of quantum neural networks, completing the inductive modeling of various security risks during the training and inference phases [4]; Debus et al. constructed a unified quantum machine learning security kill chain model, providing an engineering paradigm for standardized evaluation of attack and defense processes. However, both of these lines of work focused on the induction of risk phenomena and lacked quantitative analysis methods based on quantum physical properties [5].

Regarding privacy protection mechanisms for quantum machine learning, most existing approaches are based on the adaptation and extension of classical privacy frameworks to quantum scenarios. Watkins et al. were the first to introduce differential privacy mechanisms into the quantum learning process, using random noise to constrain information leakage [6]; Li et al. further optimized the adaptation strategy of differential privacy schemes in quantum classification networks to balance model classification accuracy and privacy protection levels [7]. However, these approaches rely on classical probabilistic privacy models and fail to leverage quantum-specific resources such as quantum entanglement and Bell’s nonlocal correlations, making it difficult to characterize the inherent privacy protection potential of quantum systems. Wang et al. proposed an integrated encoding strategy from the perspective of quantum state encoding, using circuit structure optimization to suppress training information leakage; this represents an engineering-oriented exploration focused on defensive strategies but does not establish a quantitative relationship between quantum correlations and privacy leakage risks at the fundamental physical level [8]. Heredge et al. reviewed multidimensional types of privacy leaks in quantum machine learning scenarios and established a foundational framework for privacy analysis; however, their work remained at the level of qualitative discussion and did not conduct quantitative assessments using Bell operators [9]. Ghosh et al. systematically reviewed adversarial threats and defense mechanisms in quantum machine learning, covering various paradigms such as perturbation attacks and evasion attacks; however, their review focused primarily on the quantum extensions of classical adversarial attacks and touched only briefly on intrinsic security mechanisms supported by quantum nonlocality [10].

Another line of research focuses on the noise robustness of quantum neural networks and circuit architecture optimization. Alam et al. proposed a scalable, noise-tolerant quantum neural network architecture aimed at improving the operational stability of models on NISQ devices [11]. Ahmed et al. systematically evaluated the effects of circuit depth and noise intensity on classification performance and established a robust evaluation scheme focused on task accuracy; however, this evaluation framework relies entirely on classification accuracy and overlooks the privacy and security dimensions of quantum models, suffering from the limitation of a narrow evaluation perspective [12].

The theory of quantum advantage serves as a crucial foundation for the physical analysis in this study. Huang et al. systematically reviewed the applicability boundaries of different types of quantum advantage, pointing out that quantum learning advantage exhibits significant task dependency and cannot be arbitrarily generalized [13]. Yamakawa and Zhandry provided a theoretical proof of verifiable quantum advantage without specific structural constraints, further broadening the theoretical scope of quantum advantage [14]. Kahanamoku-Meyer et al. achieved a proof of verifiable quantum advantage based on computational Bell tests, establishing a theoretical link between Bell nonlocality and quantum advantage, and providing an important theoretical basis for the analysis conducted in this paper using the CHSH inequality [15]. Daley et al. reviewed research progress on practical quantum supremacy in quantum simulation scenarios and summarized feasible pathways for achieving quantum supremacy on NISQ platforms; however, this work focused on quantum simulation tasks and did not extend to the field of privacy and security in quantum machine learning [16].

As quantum computing technology gradually penetrates high-security, sensitive fields such as privacy-preserving computing, financial risk control, medical data processing, and the management of classified government data [17], the security and trustworthiness of quantum neural networks have increasingly become a focal point of industry research. Compared to traditional artificial neural networks, variational quantum neural networks (VQNNs) leverage the parallel computing capabilities and inherent privacy-preserving properties afforded by quantum entanglement to effectively overcome the two core challenges of traditional neural networks: the computational bottleneck and privacy leakage [18]. However, their security risks exhibit a dual nature resulting from the coupling of algorithmic flaws and quantum physical characteristics; the security evaluation frameworks and protection paradigms of classical machine learning are no longer fully compatible with the intrinsic characteristics of quantum systems. Throughout the entire process of QNN forward inference and parameter iteration optimization, there are multiple security vulnerabilities that adversaries can exploit: external attackers can measure the quantum correlation statistics of circuit outputs to inversely derive the features of training samples and network parameters; by relying on a finite number of input–output queries, they can approximate and replicate the complete mapping relationship of the quantum circuit; in white-box scenarios where access to quantum hardware is available, quantum state tomography can be used to reconstruct the internal quantum states of the circuit, leading to severe risks of training data privacy leakage and model reverse engineering [19]. Therefore, identifying the physical security vulnerabilities within quantum circuits and establishing a quantized security evaluation framework tailored to the intrinsic properties of quantum systems are core research challenges that urgently need to be addressed.

Quantum entanglement is the core intrinsic correlation that distinguishes quantum systems from classical systems, and it is also the physical foundation for the potential security and computational advantages of quantum neural networks. At present, CHSH inequality testing is the mainstream method for characterizing two-body quantum nonlocal correlations and determining the presence of quantum entanglement [20].Machine learning methods have been successfully applied to the analysis of quantum system characteristics. Maroulakos et al. used classical neural networks to model quantum memory and information perturbation processes, enabling data-driven predictions of complex quantum dynamical behavior. Singh et al. further utilized artificial neural networks to automatically classify three-qubit entangled states, effectively reducing the measurement cost of identifying many-body entanglement [21,22]. More critically, quantum entanglement, Bell’s nonlocality, the expressive power of quantum computing, and cybersecurity protection capabilities are four mutually independent core concepts that do not naturally imply or derive from one another in a unidirectional manner. Most current studies suffer from serious problems of overgeneralization in their arguments; they directly equate the degree of CHSH violation with a model’s security protection capability and assume that quantum entanglement inevitably leads to privacy protection advantages. Lacking rigorous theoretical derivations and quantitative verification, the relevant conclusions exhibit obvious logical gaps and flaws in reasoning. How to precisely characterize the constraining effect of Bell’s nonlocality on local hidden-variable-based privacy-stealing attacks and establish quantitative mapping rules between quantum correlation features and privacy leakage risks are the core scientific problems that this paper urgently needs to address.

Judging from the current state of research, the security evaluation framework for quantum neural networks still has significant shortcomings. Overall, it exhibits characteristics of “classicalization, qualitative analysis, and a lack of unified standards,” which severely hampers the standardized implementation of the technology. In terms of evaluation paradigms, most existing studies directly adopt security evaluation metrics from classical neural networks, assessing security based on macro-level task performance models such as classification accuracy, prediction error, and adversarial attack success rates [23]. This approach overlooks the unique entanglement structures and nonlocal physical properties of quantum circuits, making it impossible to accurately characterize the intrinsic privacy protection capabilities of quantum models. In terms of evaluation dimensions, existing QNN security research is primarily divided into three major areas: adversarial robustness evaluation, privacy protection performance evaluation, and verification of quantum advantage; however, these areas are fragmented and lack systematic interconnections. Among these, research on adversarial robustness focuses on the design of quantum adversarial attacks and defense algorithms. It relies on algorithms such as the quantum fast gradient symbol method and quantum projected gradient descent to conduct quantitative analyses of robustness [24,25] and enhances model stability through structural optimization and adversarial training [26]. However, such research generally fails to establish a connection with the physical essence of quantum entanglement, making it difficult to explain the quantum security advantage from a fundamental mechanistic perspective [13]. Research on privacy protection relies on quantum state representation and quantum federated learning frameworks to optimize privacy protection effectiveness, using conditional entropy and data reconstruction accuracy to evaluate protection performance [27,28]. However, it has not established a quantitative link between quantum nonlocality and upper bounds on privacy leakage, and security classification standards remain ambiguous [29]. Research on quantum advantage relies on approaches such as quantum interference decoding and random circuit sampling to verify performance gains in quantum computing [30,31], yet it has failed to translate quantum advantage into an engineerable basis for security evaluation, thereby failing to support the implementation of security scenarios. Overall, the field lacks a security evaluation framework based on endogenous quantum correlations, featuring a clear adversary model, and being capable of quantitatively constraining privacy leakage.

To address these research gaps and technical shortcomings, this paper constructs a quantitative evaluation framework for the privacy security of quantum neural networks based on Bell’s nonlocal correlations. It clearly defines white-box adversary attack scenarios, focuses on local hidden-variable-type correlation-stealing attacks, and systematically investigates the privacy defense value of quantum nonlocal properties. First, this paper rigorously clarifies the boundaries of four core quantum concepts, dispelling the erroneous logical chain that entanglement exists, CHSH violation, quantum advantage, and security protection. Second, it constructs a normalized CHSH observation metric with truncation constraints, standardizing the metric’s physical meaning and scope of application to prevent overinterpretation and overgeneralization of the metric. At the same time, we define custom metrics for the relative deviation from the classical baseline accuracy and for the leakage of correlated information, which are used solely for internal cross-comparisons within the simulation scenario and are not intended for general security assessments. Building on this foundation, this paper establishes, through rigorous formal mathematical derivations, a constraint theorem relating the CHSH nonlocal observable to the upper bound on the leakage of trained privacy information, providing a quantitative proof of the suppressing effect of CHSH violation on local hidden-variable correlation-based eavesdropping attacks.

To ensure the rigor and reproducibility of the experimental results, this paper thoroughly corrects a series of issues present in the original manuscript, including conceptual confusion, statistical model inaccuracies, contradictory metric definitions, and missing experimental parameters, and supplements the full set of experimental configurations, random seeds, and training super-participant noise settings, thereby systematically enhancing the credibility of the research. It should be clarified that the numerical thresholds used in this paper serve only as reference boundaries for grouping simulation data; they are not supported by universal industry standards or universal theories and do not possess general security determination validity. The 3σ error label is merely a visual empirical benchmark and does not serve as a basis for strict statistical confidence intervals; the relevant limitations have been clearly defined in the paper.

Compared to existing research, this paper presents three innovative conclusions: First, it breaks away from the isolated evaluation model of traditional research by deeply linking the classification performance of quantum circuits with quantum nonlocality security properties, establishing a quantitative mapping relationship among degree of CHSH violation, quantum entanglement characteristics, privacy leakage risk, thereby achieving a synergistic evaluation of model task performance and security performance; second, by conducting security analysis based on observations of Bell’s inequalities under fixed measurement configurations, this study avoids the issues of decoherence and entanglement loss caused by output-state measurements, characterizes the intrinsic quantum properties of quantum neural network operations, and significantly enhances the physical validity of the security assessment; third, by establishing a quantitative evaluation framework based on the normalized CHSH metric, we overcome the limitations of qualitative judgments in traditional security analysis, achieving a breakthrough in the privacy security evaluation of quantum neural networks by transitioning from empirical analysis to quantitative deduction and providing a novel approach for constructing a security framework for quantum machine learning.

Finally, this paper systematically identifies the inherent limitations of current research: experiments were conducted via numerical simulations without physical verification on actual quantum hardware; only a single CHSH measurement basis was used, failing to comprehensively cover all quantum correlation features; the self-developed metric is limited to simulations within this group and lacks general applicability; and rigorous statistical significance tests have not yet been conducted. Based on these limitations, this paper identifies future research directions. Future work will introduce multidimensional Bell measurement schemes, experiments using actual quantum hardware, standardized model-stealing assessment paradigms, and rigorous statistical testing methods to continuously refine the quantitative security evaluation framework for quantum neural networks and advance the standardization and engineering development of quantum machine learning security technologies.

The subsequent chapters of this paper are organized as follows: Section 2 provides a systematic introduction to fundamental theoretical concepts such as quantum entanglement and Bell’s inequalities;Section 3 details the definition of measurement metrics, error handling methods, and the security quantification framework; Section 4 comprehensively describes dataset preprocessing, model architecture, and simulation experiment configurations; Section 5 analyzes simulation results, verifies the patterns of the metrics, and discusses the limitations of the study; and Section 6 summarizes the research content of the entire paper and outlines future research directions.

## 2. Related Theories

### 2.1. Quantum Entanglement

Quantum entanglement was first proposed by Einstein, Podolsky, and Rosen [32] and Schrödinger [33]. It was once regarded as a counterintuitive and peculiar phenomenon in quantum mechanics, sparking extensive debate regarding the completeness of quantum theory. Subsequently, Bell rigorously proved that quantum entanglement leads to a significant, experimentally testable deviation between quantum mechanics and classical physics [34], laying a crucial theoretical foundation for the experimental verification of entanglement. With the rise of quantum information science, quantum entanglement has been widely recognized as a core resource for quantum information processing, underpinning the realization of key technologies such as quantum cryptography [35], quantum teleportation [36], and measurement-based quantum computing [37]. With the rapid development of quantum control experimental techniques, academic research into the fundamental theory of quantum entanglement has continued to deepen, and the preparation and verification of various quantum entangled states have become one of the core directions of current experimental research.

In recent years, machine learning has become an important tool for analyzing the characteristics of quantum correlations. Maroulakos et al. [21] used classical neural networks to study the dynamics of quantum memory and information perturbation, demonstrating that data-driven methods can characterize the evolution of complex quantum systems and provide feasible observational approaches for quantum dynamical problems that are difficult to solve analytically. Singh et al. [22] used artificial neural networks to classify the entanglement of arbitrary three-qubit quantum states, distinguishing between different types of many-body entanglement based on the SLOCC criterion, thereby significantly reducing the measurement resources required for many-body entanglement identification. The above work confirms that neural networks possess the ability to extract quantum correlation features; however, their scope is limited to quantum dynamical evolution and quantum state identification. They have not established a quantitative link between quantum entanglement, nonlocality, and model privacy leakage, and therefore cannot be directly applied to the security assessment of quantum neural networks.

### 2.2. Bell’s Inequality

**Figure 1:**
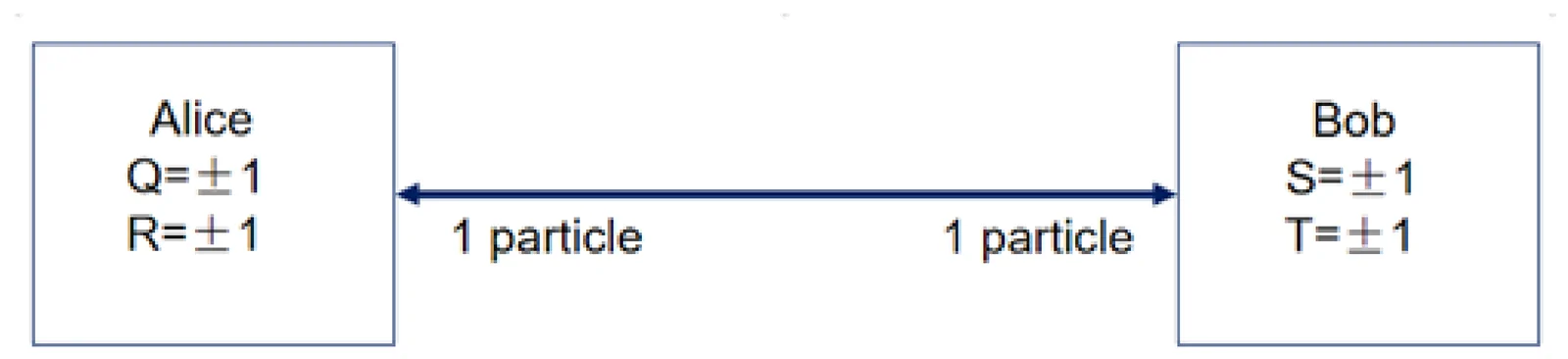
The overall experimental setup for the Bell inequality experiment.

We can envision conducting the experiment shown in Figure 1: Charlie is responsible for preparing a pair of particles. The specific method he uses to prepare the particles is irrelevant; what matters is that he can reliably repeat the preparation process to obtain reproducible experimental samples. Once the particles are prepared, Charlie sends one particle to Alice and the other to Bob, laying the groundwork for subsequent quantum entanglement verification and measurement experiments.

In the experiment, Alice can choose to perform either a *Q* or an *R* measurement on the particle, while Bob can choose to perform either an *S* or a *T* measurement; both parties complete their measurements simultaneously. Since Alice and Bob are sufficiently far apart, their measurement operations are independent of one another and do not interfere with each other.

As soon as Alice receives the particle, she immediately measures it. Suppose Alice has two different measurement devices at her disposal, allowing her to choose between two distinct measurement schemes, whose physical properties are denoted as $P_{Q}$ and $P_{R}$, respectively. It is important to note that Alice does not know which measurement scheme she will use before receiving the particle; instead, she determines the final measurement scheme by flipping a coin after receiving the particle. To simplify the analysis, assume that each measurement method yields only two possible results: +1 and −1. Let *Q* denote the value of the particle measured by Alice under the $P_{Q}$ measurement method; this *Q* value is defined as the intrinsic objective property of the particle, determined in the same way that the position of a tennis ball is determined by the light particles it emits. Similarly, let *R* denote the measurement result obtained by Alice under the $P_{R}$ measurement method.

Similarly, let Bob also be able to measure the particle he receives, specifically choosing to measure either the $P_{S}$ or $P_{T}$ property. After measurement, he obtains the objective value of the corresponding physical quantity, that is, the measured value corresponding to the particle’s intrinsic properties *S* and *T*, both of which are strictly limited to +1 or −1. Bob does not need to determine in advance which property to measure; instead, after receiving the particle, he randomly selects either $P_{S}$ or $P_{T}$ to measure. Similarly, Alice may also randomly select the measurement target. To eliminate mutual interference between measurements, Alice and Bob must conduct their measurements at exactly the same moment; more rigorously stated from a relativistic perspective, there is no causal relationship between their measurement actions. Based on the principles of relativity, the propagation speed of physical interactions cannot exceed the speed of light. Therefore, Alice’s measurement cannot possibly interfere with Bob’s measurement results (and vice versa). This is because the propagation speed of any physical interaction is bound by the speed-of-light limit, thereby ensuring that the two types of measurement are independent of each other and free from causal interference.

Next, we will perform a simple algebraic derivation and calculation for the physical quantity QS+RS+RT−QT. Please note that

(1) QS+RS+RT−QT=(Q+R)S+(R−Q)T

Given the condition R,Q=±1, the system can be in one of two states: (Q+R)S=0 or (R−Q)T=0. It is easy to see from Equation (1) that for each case, QS+RS+RT−QT=±2; let us assume that the probabilities that the system is in states Q=q,R=r,S=s and T=t before measurement are p(q,r,s,t), respectively. These probabilities may depend on Charlie’s method of particle preparation and may also be affected by experimental noise. Let E(⋅) denote the mean of the physical quantity to be measured; then, we have

(2) $$\begin{array}{c} E\left(QS+RS+RT-QT\right)=\sum_{qrst}p(q,r,s,t)\left(qs+rs+rt-qt\right)\\ \leq \sum_{qrst}p(q,r,s,t)\times 2\\ =2 \end{array}$$

and

(3) $$\begin{array}{c} E\left(QS+RS+RT-QT\right)=\sum_{qrst}p(q,r,s,t)qs+\sum_{qrst}p(q,r,s,t)rs\\ +\sum_{qrst}p(q,r,s,t)rt-\sum_{qrst}p(q,r,s,t)qt)\\ =E(QS)+E(RS)+E(RT)-E(QT) \end{array}$$

Comparing Equations (2) and (3), we obtain Bell’s inequality,

(4) E(QS)+E(RS)+E(RT)−E(QT)≤2

This inequality is also commonly referred to as the CHSH inequality.

Let us connect these ideas to quantum physics. Assuming that Alice and Bob both have a Quantum mechanical spin-1/2 particle, one can identify $A_{i}$ and $B_{j}$ with the measurement results of the Pauli spin operators and $Y=\sigma_{y}$, respectively. Then, the left-hand side of Equation (4) defines a quantum mechanical observable $E=A_{1}⊗B_{1}+A_{2}⊗B_{1}+A_{1}⊗B_{2}-A_{2}⊗B_{2}$, the so-called Bell operator, that can be measured. One finds that there are quantum states that violate the inequality and give $2\sqrt{2}$ for the left-hand side. Taking the observables $A_{1}=-\sigma_{x}$, $A_{2}=-\sigma_{y}$, $B_{1}=\left(\sigma_{x}+\sigma_{y}\right)/\sqrt{2}\;$ and $B_{2}=\left(\sigma_{x}-\sigma_{y}\right)/\sqrt{2}\;$ the quantum state with the highest violation is the eigenstate corresponding to the largest eigenvalue of this Bell operator. It is given by

(5) $$|\psi_{CHSH}\rangle =\frac{1}{\sqrt{2}}\left(|01\rangle -|10\rangle \right)$$

which is just the two-qubit singlet state. It can be shown that quantum mechanics does not allow for violations larger than $2\sqrt{2}$, a fact which is known as the Tsirelson bound.

### 2.3. Violation of the CHSH Inequality and Entanglement

The violation of a Bell inequality implies nonlocality, i.e., that the measurement probabilities cannot be described by a local hidden variable (LHV) model. In quantum mechanics, the violation implies that the state is entangled. This can be seen as follows. By construction, a separable state in a bipartite system can be written in the form $\phi =\sum_{k}p_{k}\phi_{k}^{A}⊗\phi_{k}^{B}$. Hence, one can write

(6) $$\langle A_{i}B_{j}\rangle =\sum_{k}p_{k}Tr\left(A_{i}\phi_{k}^{A}\right)Tr\left(B_{j}\phi_{k}^{B}\right)$$

which is, if we define $A_{k}\left(A_{i}\right)=Tr\left(A_{i}\phi_{k}^{A}\right)$ and $B_{k}\left(B_{j}\right)=Tr\left(B_{j}\phi_{k}^{B}\right)$, directly a LHV model. This means that if some measurements on a state violate a Bell inequality, then it cannot be separable, i.e., it must be entangled. Note, however, that the converse is not true: there are entangled states for which a local realistic model for all von Neumann measurements can be written down explicitly (see also below). Therefore, they do not violate any Bell inequality. The same statement holds even if one allows positive-operator-valued measurements (POVMs). For pure states, however, any bipartite entangled state violates a Bell inequality. Due to that, Bell inequalities may be viewed as non-optimal entanglement witnesses.

Finally, before considering further Bell inequalities, let us mention ways to characterize them from the point of view of their usefulness in experiments. We have seen that the CHSH inequality is maximally violated by the state Equation (5). However, in a real experiment, one can never prepare the state Equation (5) perfectly; it is always mixed with noise

(7) $$\phi_{noisy\sin glet}\left(p\right)=|\psi_{CHSH}\rangle \langle \psi_{CHSH}|+\left(1-P\right)\frac{I}{4}$$

which is a two-qubit Werner state [38]. The CHSH inequality is violated if $p>1/\sqrt{2}\;\approx 0.71$ [39] and, using the positive partial transposition PPT criterion, the state is entangled for p>1/3. Moreover, it is known that this state allows for an LHV model (for all von Neumann measurements) for p<0.6595, and it violates a Bell inequality (different from the CHSH one) for p>0.7056.

The bound for the CHSH inequality characterizes the minimum visibility that the CHSH inequality requires. In general, the visibility is the ratio between the maximum of the Bell operator *B* for quantum states and the maximum for LHV models.

(8) $$V\left(B\right)=\frac{\max_{\psi }\left|{\langle B\rangle }_{\psi }\right|}{\max_{LHV}\left|{\langle B\rangle }_{LHV}\right|}$$

Clearly, a large visibility is an advantage from the experimental point of view.

Furthermore, this paper strictly distinguishes between four mutually independent concepts: quantum entanglement, Bell’s nonlocality, quantum computational advantage, and quantum cybersecurity. (1) Quantum entanglement is an intrinsic correlation inherent to quantum states; Bell’s nonlocality describes the fact that measurement statistics cannot be explained by local hidden-variable models; the existence of entanglement does not necessarily imply that Bell’s nonlocal correlations can be observed. (2) The ability to observe Bell nonlocality does not imply that a variational quantum circuit possesses quantum computational advantage. (3) Even if a circuit possesses quantum computational advantage, this does not directly imply security capabilities such as training set privacy protection or resistance to model theft.

## 3. Preliminary Preparations

Based on the research framework established in the preceding sections, this paper addresses the shortcomings of existing research by refining and supplementing the research content and improving the research framework in areas such as QNN parameter initialization, CHSH inequality error calibration, and the definition of secure quantization metrics. The specific optimization directions are as follows:

### 3.1. Initialization of QNN Circuit Parameters

The circuit parameter initialization strategy for quantum neural networks (QNNs) directly determines the convergence efficiency of model training, the stability of gradient evolution, and the final classification performance. To balance the effectiveness of quantum data encoding with the stability of the model training process, this study employs a hierarchical initialization scheme. This approach effectively suppresses gradient vanishing and avoids local optima while ensuring the expressive power of quantum feature mapping and parameter learnability, thereby guaranteeing the stable training and optimal performance of QNN models.

#### 3.1.1. Data Encoding Layer Parameters

The data encoding layer employs the RY angle encoding scheme, which essentially maps classical data, after normalization, to the rotation angles of qubits. This enables the efficient and lossless embedding of classical features into quantum states, providing reliable input for the subsequent training of variational quantum circuits. The Iris dataset’s 4-dimensional continuous features are defined as follows:

(9) $$\begin{array}{c} x_{1}:SepalLength,x_{2}:SepalWidth\\ x_{3}:PetalLength,x_{4}:PetalWidth \end{array}$$

During the data preprocessing stage, a min-max normalization method is employed to uniformly map all raw features to the [0, 1] interval. The normalization formula is: $\theta_{i}=\frac{x_{i}-x_{\min,i}}{x_{\max,i}-x_{\min,i}}\times 2\pi ,\left(i=1,2,3,4\right)$. The normalized features are converted into the effective rotation angles of the quantum RY rotation gate via a predefined mapping relationship and encoded into the corresponding quantum circuits in sequence. This ensures that quantum state preparation remains within the theoretically feasible range, effectively enhancing encoding stability and model training convergence. This RY angle encoding method combines the advantages of clear physical meaning, stable quantum state preparation, and no redundant parameters. It efficiently achieves a lossless conversion from classical features to quantum states, providing high-quality input support for the subsequent variational quantum circuit learning process.

This study employs a 2-qubit encoding circuit to validate fundamental quantum encoding logic, using the RY rotation gate as the core encoding operation unit. First, classical features undergo normalization and angle mapping, converting feature values into rotation parameters compatible with quantum gates; subsequently, these parameters are sequentially loaded onto qubit 0 and qubit 1, completing the encoding of classical information into a quantum state. After encoding, a CNOT entanglement gate is used to establish correlations between qubits, providing a foundational circuit framework for subsequent quantum feature learning, model training, and verification of quantum entanglement properties.

#### 3.1.2. Entanglement Layer Parameters

The entanglement layer contains four trainable parameters $\left(\alpha_{1},\alpha_{2},\beta_{1},\beta_{2}\right)$. Its core function is to establish correlations and feature couplings among qubits, making it a critical component for enhancing the expressive power of QNN models. To avoid issues such as limited model representational capacity caused by overly concentrated parameter distributions and the tendency for the training process to get stuck in local optima, this study employs a uniform distribution initialization strategy $\alpha_{1},\alpha_{2}∼U\left(0,\pi /2\;\right)$, $\beta_{1},\beta_{2}∼U\left(\pi /2\;,\pi \right)$. This ensures that the trainable parameters fully cover the sensitive intervals of quantum rotation gates, guarantees the diversity of quantum states during the early training phase, and ensures the stability of the gradient descent process, thereby laying the foundation for efficient model training. The entanglement layer’s quantum circuit is directly connected to the encoding layer. Through a cascaded structure of parameterized rotation gates and entanglement gates, it effectively establishes quantum correlations among multiple qubits and facilitates deep feature interaction, further enhancing the model’s nonlinear representation capabilities and providing robust support for the accurate execution of subsequent classification tasks.

#### 3.1.3. Measurement Layer Design

The measurement layer combines a two-qubit basis measurement with a probability normalization strategy to accommodate the three-class classification task requirements of the Iris dataset. Specifically, *Z*-basis $\sigma_{z}$ measurements are performed on qubits $q_{0}$ and $q_{1}$ to obtain the output probability distribution $P_{0},P_{1},P_{2},P_{3}$ corresponding to the four measurement outcomes $\left(|00\rangle ,|01\rangle ,|10\rangle ,|11\rangle \right)$. Subsequently, a predefined mapping rule is applied to compress the 4-dimensional probability vector into a 3-dimensional classification score, thereby achieving a stable conversion from quantum measurement results to classical classification labels and ensuring the validity and robustness of the classification output. The formula is as follows:

(10) $$\left\{\begin{array}{c} s_{0}=P_{0}+P_{1}/2\to (Setosa)\;\\ s_{1}=P_{1}/2\;+P_{2}\to \left(Versicolor\right)\\ s_{2}=P_{2}/2\;+P_{3}\to \left(Virginica\right) \end{array}\right.$$

### 3.2. Error Calibration Methods for CHSH Inequality Verification

The CHSH inequality test is a core technical method for determining the presence of quantum entanglement, but its computational results are susceptible to dual interference from measurement statistical errors and quantum decoherence effects, the former arising from statistical fluctuations during the measurement process, and the latter from the natural decay of quantum states. Together, these factors cause the observed CHSH values to deviate from the true values, thereby affecting the accuracy of entanglement detection. To ensure the rigor of quantum entanglement detection and guarantee the reliability of verification results, this study employs a combined error suppression strategy of sample size optimization and confidence interval calibration. This approach effectively mitigates the dual interference, enabling precise estimation and correction of CHSH values and providing reliable support for the scientific determination of entanglement.

#### 3.2.1. Calculation of the Basic CHSH Value

First, define the correlation function of the intermediate state in the QNN: For the intermediate quantum state $\rho_{mid}$ evolved from the QNN, set the measurement basis at Alice’s ($q_{0}$) end to $a_{1}=\sigma_{x},a_{2}=\sigma_{z}$ and the measurement basis at Bob’s ($q_{1}$) end to $b_{1}=\left(\sigma_{x}+\sigma_{z}\right)/\sqrt{2}\;,b_{2}=\left(\sigma_{x}-\sigma_{z}\right)/\sqrt{2}\;$, thereby constructing the correlation function $E\left(a_{i},b_{j}\right)=Tr\left[\rho_{mid}\left(a_{i}⊗b_{j}\right)\right]$. Based on this, the CHSH value can be expressed as $S=\left|E\left(a_{1},b_{1}\right)-E\left(a_{1},b_{2}\right)+E\left(a_{2},b_{1}\right)+E\left(a_{2},b_{2}\right)\right|$, with a corresponding classical limit of S≤2 and a quantum mechanical theoretical upper bound of $S\leq 2\sqrt{2}\approx 2.828$. CHSH values that have not undergone error calibration are calculated solely based on raw measurement statistics and are susceptible to interference from factors such as a limited number of samples and system decoherence. Consequently, the results are subject to bias and cannot be directly used for the rigorous determination of quantum entanglement.

#### 3.2.2. Error Calibration Strategy

To enhance the reliability and statistical confidence of the Bell inequality test, this study employs a dual-optimization strategy combining statistical error calibration with systematic error suppression. This approach rigorously corrects and controls measurement noise, quantum state evolution noise, and statistical fluctuations during the experiment, effectively reducing the interference of various errors on experimental results. It ensures that the determination of quantum entanglement is accurate, reliable, and reproducible, thereby providing rigorous data support for QNN quantum security analysis.

The statistical error σ in a measurement is directly determined by the number of measurements $n_{shots}$ and can be ensured through standardized calibration to guarantee the reliability of the experimental results.

Single-correlation-function error: A statistical estimate is made of the standard deviation $\sigma =\sqrt{\frac{1-E^{2}}{n_{shots}}}$ of a single correlation function *E*. This standard deviation quantitatively reflects the level of statistical fluctuations during a single measurement, providing a foundation for error traceability.

Total Error Synthesis: The total composite error $\sigma_{S}=\sqrt{\sigma_{E1}^{2}+\sigma_{E2}^{2}+\sigma_{E3}^{2}+\sigma_{E4}^{2}}$ of the CHSH operator is obtained through orthogonal synthesis of the statistical errors of the four correlation functions. Specifically, this is calculated by taking the square root of the sum of the squares of the statistical errors of the four correlation functions, thereby achieving precise synthesis and integration of multi-source measurement errors.

### 3.3. Definition of Quantifiable Security Metrics

Based on the degree of violation of the CHSH inequality, this paper defines three quantifiable quantum security metrics, establishing a complete mathematical mapping relationship between quantum entanglement properties and security performance. This provides a solid theoretical foundation and quantitative basis for the scientific assessment of the security levels of quantum neural networks (QNNs).

Security Analysis: Upper Bounds on Privacy Leakage Constraints Based on the CHSH Nonlocality Criterion.

Existing work often intuitively applies quantum nonlocality to security discussions, but this can lead to logical leaps; simply observing a violation of the CHSH inequality does not directly imply that a quantum neural network possesses general security capabilities. A violation of the CHSH inequality merely rules out a local hidden-variable explanation for measurement correlations; training privacy, model-stealing resistance, and adversarial robustness are mutually independent security properties that require clear definitions of the adversary model and quantitative metrics.

This paper establishes a formal constraint: under the white-box adversary assumption, where the variational circuit is known and the encoded quantum state can be replicated, we define the maximum training sample mutual information $I_{LV}\left(X;M\right)$ that an adversary can extract using a local hidden variable strategy. The paper proves the existence of a monotonically decreasing function $f\left(\cdot \right)$ such that

(11) $$I_{LV}\left(X;M\right)\leq f\left(S\right),$$

where *S* is the CHSH expectation. The stronger the CHSH violation, the lower the correlation capacity that a local hidden-variable model can support, and the upper bound on the amount of training privacy that an adversary can steal using such strategies is compressed (the complete proof is moved to Appendix A).

At the same time, the scope of the argument must be clearly defined: First, this theorem only constrains correlation-stealing attacks under the local hidden-variable paradigm; it cannot prevent the reverse reconstruction of quantum states achieved through quantum tomography or repeated state preparation. Second, the input in this paper consists of classically encoded data, and the adversary can know the complete circuit structure; the attacker does not need to clone an unknown single-qubit state to carry out a query attack. Third, the no-cloning theorem protects unknown single-quantum states but cannot defend against measurement tomography following multiple preparations of the same encoded state. Fourth, this conclusion cannot be generalized to nonlocal hidden-variable threats such as adversarial sample attacks or man-in-the-middle attacks; the security properties against these threats require independent research.

In summary, CHSH nonlocal correlations can serve as a quantum physical resource to mitigate a certain class of privacy-stealing attacks, but they cannot stand alone as a universal security guarantee for quantum neural networks. Establishing unified security criteria across different attack types is proposed as an open research direction for the future.

#### 3.3.1. CHSH Normalized Observation Indicators (Q)

a. Definition and Physical Significance of CHSH Normalized Observation Indicators (Q)

This paper adds a truncation constraint to the outer layer of the *Q* definition:

(12) $$Q=\min(0,\max\left(1,\frac{S-2}{2\sqrt{2}-2}\right))$$

It is explicitly stated that if $S>2\sqrt{2}$ after calibration, Q=1 is strictly limited; if S<2, *Q* is set to 0 to ensure strict mathematical compliance with $Q\in \left[0,1\right]$. The inherent limitations of the calibration method are also discussed, and it is recommended that future research optimize noise compensation strategies. Its core physical significance lies in characterizing the extent to which the internal quantum state of a QNN deviates from the limits of classical local theory; it serves as a key metric for quantifying the strength of true quantum entanglement. *Q* is merely a normalized observation of the degree of CHSH violation under a given measurement configuration; it is not a universal measure of entanglement, nor is it an indicator of quantum computational advantage, nor can it serve independently as a standard for evaluating security performance. Specifically, *Q* only quantifies the degree to which nonlocal correlations manifest under this set of measurements; it cannot be used to compare the total amount of entanglement across different measurement schemes or circuit architectures, nor can it directly characterize a model’s expressiveness, advantages, or security. In the security theorems discussed below, it is treated solely as an observed input parameter under a specified measurement scheme.

The details are shown in Table 1. Its core physical significance lies in characterizing the extent to which the internal quantum state of a QNN deviates from the classical local theory limit. It serves as a key metric for quantifying the strength of true quantum entanglement and is directly related to the quantum security performance of the QNN.

b. Security Group Reference Thresholds

Based on the magnitude of the CHSH-normalized observation metric Q, clearly define the reference thresholds for QNN security grouping to achieve a quantitative definition of security performance, as shown in Table 2. The values 0.3 and 0.7 serve solely as reference thresholds for dividing the experimental groups in the numerical simulations presented in this paper and are used exclusively for horizontal comparisons of the results within this study. It is explicitly stated that there is no supporting general theory, risk model, or calibration dataset, and these results cannot be directly applied to other quantum circuits, measurement configurations, or attack scenarios.

#### 3.3.2. Relative Deviation of Classic Baseline Replicas (Δ)

a. Metric Definition

We construct the $\Delta =1-\frac{Acc_{classical}}{Acc_{QNN}}$ metric to quantify how difficult it is for optimal classical classification models to approach the performance of quantum neural networks in terms of task-label accuracy. This metric does not formally characterize the similarity between the output distributions of replicated models and those of the original QNN under black-box or white-box model-stealing attacks; it is used solely to compare the gap between optimal classical classification baselines and QNNs in terms of task-label accuracy, serving as a reference for classical simulation capabilities at the task level, and is not equivalent to model-stealing attack metrics. The core parameters are defined as follows: $Acc_{QNN}$: The classification accuracy of the quantum neural network (QNN) itself; $Acc_{classical}$: The classification accuracy on a unified test set achieved by classical machine learning models (represented by Random Forest) after replicating the QNN’s decision boundary. The metric quantifies the ability of classical models to replicate the QNN’s classification logic based on the difference between $Acc_{QNN}$ and $Acc_{classical}$.

b. Physical and Engineering Significance

The core value of this metric lies in its ability to quantitatively characterize the difficulty for classical machine learning models to replicate the intrinsic classification logic and quantum feature mapping patterns of QNNs. In particular, a higher Δ value indicates that it is more difficult for a classical model to approximate the decision boundary of a QNN and replicate its quantum correlation properties, implying that the QNN offers stronger privacy protection; Δ=0 indicates that a classical model can achieve the same classification accuracy, meaning that the network’s behavior at this task level is easier for a classical model to simulate. This metric is used solely as an auxiliary observation for cross-comparison in the experiments of this paper.

c. Specific Calculation Process

Model Inference: Using the trained QNN model, perform inference and prediction on the Iris dataset to output the model’s predicted label $y_{qnn}$.

Classical Model Training: Using the original feature data *X* from the Iris dataset as input and the QNN predicted label $y_{qnn}$ as output, construct a training set to train a classical Random Forest model, simulating the process by which classical algorithms attempt to replicate the QNN’s decision boundary.

Metric Calculation: Calculate the classification accuracy $Acc_{classical}$ of the Random Forest model on a unified test set. Substitute $Acc_{QNN}$ and $Acc_{classical}$ into the predefined metric formula to solve for the classical replication error Δ, thereby completing the quantitative evaluation of the QNN’s resistance to classical replication.

#### 3.3.3. Privacy Protection Strength (*P*)

The privacy protection strength P=Q×(1−ε) is jointly constructed by combining the observation metric *Q* with the normalized exposure coefficient ε that can be extracted from externally correlated observations based on the training data. The smaller the value of ε, the stricter the privacy protection constraints and the lower the risk of data exposure.

This metric is constructed based on both quantum physical properties and statistical security theory and is primarily used to quantify the comprehensive privacy protection capabilities of QNNs within a federated learning framework. Specifically, the observation metric *Q* leverages the inherent physical property of quantum nonlocality to suppress the reverse leakage of model gradient information at the fundamental physical level, thereby forming a natural quantum-level privacy barrier. The ε metric characterizes the relative level to which external observation channels can extract training-related information. The two metrics work in tandem, complementing and reinforcing each other to achieve dual privacy protection, physical-level resistance to replication and statistical-level protection against leakage, ensuring the scientific rigor and practical applicability of the metric.

The value of Privacy Protection P=Q×(1−ε) is strictly confined to the theoretical range $\left[0,1\right]$, with the following grading criteria: when P≥0.5, the QNN model is deemed to possess robust and effective privacy protection capabilities and can be directly applied to application scenarios with extremely high privacy security requirements, such as privacy-preserving computing, distributed government data collaboration, and modeling of sensitive medical data.

## 4. Experimental Framework Design

This study deeply integrates the Iris classification task for quantum neural networks (QNNs) with Bell’s inequality verification methods to establish a systematic and quantifiable research framework for QNN security. The core logic is as follows: use the Iris classification task as a practical application and rely on Bell’s inequality, the core criterion for determining quantum entanglement, to determine whether the quantum entanglement generated during the QNN classification process constitutes genuine quantum correlation and whether it can withstand counterfeiting by classical algorithms, thereby quantitatively defining the security boundaries and security levels of QNNs. In this context, genuine quantum entanglement serves as the core quantum resource enabling QNNs to effectively resist reverse engineering by classical algorithms and ensure the privacy and integrity of classification results. It is also the key prerequisite and fundamental basis for QNNs to achieve quantum security advantages. The following sections will detail the complete research framework, specific implementation steps, and key verification dimensions, clarifying the logical relationships and implementation priorities at each stage to ensure that the entire research process is rigorous, scientifically sound, reproducible, and practical.

### 4.1. Core Logic of the Study: The Foundation of Coupling Tasks and Verification

The core of this research lies in the organic integration of the Iris classification task with the verification of Bell’s inequality, featuring a clear and rigorous underlying logic; as a standard benchmark task in the field of classical machine learning, Iris classification involves three classification labels and four-dimensional features (sepals length/width; petals length/width), which can be modeled quantum mechanically using parameterized quantum circuits (PQCs), specifically, by encoding classical features into quantum states and then using entanglement gates such as CNOT and SWAP to establish higher-order correlations among features, ultimately accomplishing the classification task. In this process, quantum entanglement serves as the core physical resource for enhancing the expressive power of QNN models and enabling efficient classification, directly determining whether the model can transcend the limitations of classical feature representation. As the core scientific criterion for verifying quantum entanglement, Bell’s inequality plays a pivotal role in verifying whether the quantum states generated during QNN evolution exceed the limits of classical local theory, thereby precisely distinguishing true quantum entanglement from classically simulable pseudo-entanglement. The core security significance of this combination of tasks and verification methods lies in the following: if a QNN generates only pseudo-entanglement and fails to form genuine quantum nonlocal correlations, its classification logic can be easily replicated and reverse-engineered by classical algorithms, offering no quantum security advantages; if the QNN generates true quantum entanglement, then based on the quantum no-cloning theorem and quantum nonlocality, its model structure, classification logic, and input data will inherently possess unique security properties that are resistant to classical forgery and eavesdropping; this is also the core premise for this study’s exploration of the QNN’s quantum security advantages.

### 4.2. Research Framework: The Complete Process from QNN Classification to Bell’s Inequality Verification

Phase 1: Building a QNN Model for Iris Classification: Implementation of the Base Task

1. Standard data preprocessing

This study selected the Iris dataset as the experimental task carrier. This dataset contains four continuous features (sepal length, sepal width, petal length, and petal width) and three classification labels. It is a standard benchmark dataset widely used in the field of classical machine learning, characterized by a clear data structure and well-defined classifiability, making it suitable for verifying the basic functionality and security properties of QNN models. To adapt to the input requirements of quantum circuits, ensure that classical features can be effectively mapped to quantum states, and avoid encoding distortion, the dataset undergoes the following preprocessing:

The min-max normalization method was employed to uniformly scale the 4-dimensional classical features to a specified range $\left[0,2\pi \right]$, ensuring that the numerical range of the features precisely matches the angular range of quantum rotation gates (such as RY and RZ gates). This provides standardized input for subsequent quantum state mapping, ensuring the stability and accuracy of quantum circuit evolution.

An angle-encoding strategy is employed to map the normalized 4-dimensional features to rotation gate operations on two qubits ($q_{0}$ and $q_{1}$). The specific mapping rules are as follows: the angle of the RY gate for $q_{0}$ is assigned by feature 1, the angle of the RY gate for $q_{1}$ is assigned by feature 2, the angle of the RZ gate for $q_{0}$ is assigned by feature 3, and the angle of $q_{1}$’s gate is determined by feature 4, ultimately constructing the initial quantum state $|\psi_{input}\rangle$ that satisfies the requirements for quantum circuit evolution.

2. QNN Model Design

The design involves a parameterized quantum circuit (PQC) comprising a feature encoding layer, a quantum entanglement layer, and a measurement output layer. The core structure balances the implementation of classification functions with the requirements for quantum security verification, ensuring both the model’s classification performance and the reservation of a dedicated interface for Bell’s inequality verification. The specific design is as follows:

At least two qubits (denoted as $q_{0}$ and $q_{1}$) are selected. Hardware and logical interfaces are reserved for subsequent Bell inequality verification, which requires a two-qubit entangled system, ensuring that the verification phase can be directly integrated without requiring additional adjustments to the circuit architecture.

A three-tier integrated encoding-entanglement-measurement architecture is adopted. This design ensures the accuracy requirements of the classification task through a reasonable hierarchical structure while also providing a solid physical and circuit foundation for the quantitative analysis of quantum security performance (Bell inequality verification and quantum entanglement determination).

RY angle encoding is utilized to map the four-dimensional features of the Iris flower ($\theta_{1}∼\theta_{4}$ sepal length, sepal width, petal length, and petal width) to the rotation gates $RY\left(\theta_{1}\right)⊗RY\left(\theta_{2}\right)+RZ\left(\theta_{3}\right)⊗RZ\left(\theta_{4}\right)$ of $q_{0}$ and $q_{1}$, respectively. This achieves a lossless conversion from classical features to quantum states, ensuring the effective transmission of feature information.

Due to the quantum entanglement layer, $CNOT\left(q_{0},q_{1}\right)+RY\left(\alpha \right)⊗RY\left(\beta \right)+CNOT\left(q_{1},q_{0}\right)$ and by introducing trainable entanglement gates (parameters denoted as α and β), the entanglement strength between qubits is adjusted to enhance the model’s expressive power. Simultaneously, this provides a detectable entanglement carrier for subsequent Bell inequality verification, achieving a synergistic improvement in both classification performance and security performance.

It performs $\sigma_{-}z$ basis measurements on the two qubits $q_{0}$ and $q_{1}$, respectively, and maps the quantum state results to classical probability distributions, ultimately converting them into Iris classification results, e.g., Setosa/Versicolor/Virginica.

The trainable parameters (α, β) of the entanglement layer are optimized using a gradient descent algorithm to minimize the classification cross-entropy loss. The goal is to achieve an Iris classification accuracy of over 95% (classical machine learning models can achieve up to 98% accuracy). Simultaneously, by adjusting the entanglement layer parameters, a stable quantum state foundation is provided for subsequent Bell inequality verification, thereby achieving dual compliance with both classification performance and security performance.

Phase 2: Embedding the Bell’s Inequality Verification Module

The core innovation of this study lies in the precise embedding of a Bell’s inequality verification module throughout the entire QNN classification process, which constitutes the core innovation of this research. Unlike traditional QNN research paradigms that focus solely on classification results, this study systematically verifies the authenticity and stability of QNN quantum entanglement from the dual dimensions of process entanglement and result entanglement by embedding Bell’s inequality verification nodes throughout the entire QNN classification process. This provides a core, reliable quantitative basis for subsequent QNN security assessments. The specific verification process is as follows:

a. Verification Node 1

Verification of Bell’s inequality violations in QNN intermediate states, which is a critical step in determining entanglement generation. Embedding location: Precisely deployed between the completion of the QNN entanglement layer, e.g., CNOT, SWAP, and other entanglement gates and the initiation of the measurement layer, simultaneously extracting the instantaneous quantum states $\rho_{mid}$ of the two qubits $q_{0}$ and $q_{1}$ at this node, that is, the intermediate quantum state formed after the entanglement gates have acted, to serve as the core sample for verifying whether the QNN has generated genuine entanglement.

Verification method (CHSH inequality verification): ① Perform four sets of orthogonal measurements on the intermediate quantum state $\rho_{mid}$, corresponding to the measurement bases of Alice and Bob ($\sigma_{x}$ and $\sigma_{-}z$), and calculate the correlation function $E(a_{i},b_{j})$ for each set of measurements. ② Calculate the CHSH value $S=\left|E\left(a_{1},b_{1}\right)-E\left(a_{1},b_{2}\right)+E\left(a_{2},b_{1}\right)+E\left(a_{2},b_{2}\right)\right|$ based on the correlation function $E(a_{i},b_{j})$. ③ Decision criterion: If s>2+3σ (where σ represents statistical error), the intermediate quantum state $\rho_{mid}$ is determined to contain genuine quantum entanglement.

b. Verification Node 2: Violation of Bell’s Inequalities by the QNN Output State

Implementation Point: After the QNN completes classification measurements and outputs classification results, perform Bell’s inequality verification on the output mixed state $\rho_{output}$, which carries the classification label. Special Processing: Considering that the output state is a post-measurement mixed state subject to quantum state distortion, quantum tomography must first be employed to reconstruct the output mixed state $\rho_{output}$, effectively eliminating interference introduced by the measurement process; Subsequently, CHSH values are calculated based on the reconstructed quantum state to verify whether quantum entanglement properties are effectively preserved through to the output stage, where a higher degree of entanglement preservation indicates stronger security characteristics of the QNN classification results, such as privacy protection and resistance to classical attacks.

Phase 3: Security Analysis

Based on the results of the Bell inequality verification described above, this study systematically analyzes the security performance of the QNN Iris classification task from three key dimensions, clarifying the intrinsic relationship between quantum entanglement and the security properties of QNNs. The specific analysis is shown in Table 3 below.

## 5. Safety Verification and Assessment

This experiment focuses on the quantum security properties of quantum neural networks (QNNs). By integrating experimental data from various groups, results from Bell’s inequality tests, and various quantitative metrics of quantum security, we conduct a comprehensive, systematic, and rigorous analysis of the QNN’s quantum security properties across three core dimensions: the authenticity of quantum entanglement, the quantitative performance of security, and the reliability of experimental data. This approach ensures the scientific validity and credibility of our conclusions.

**Figure 2:**
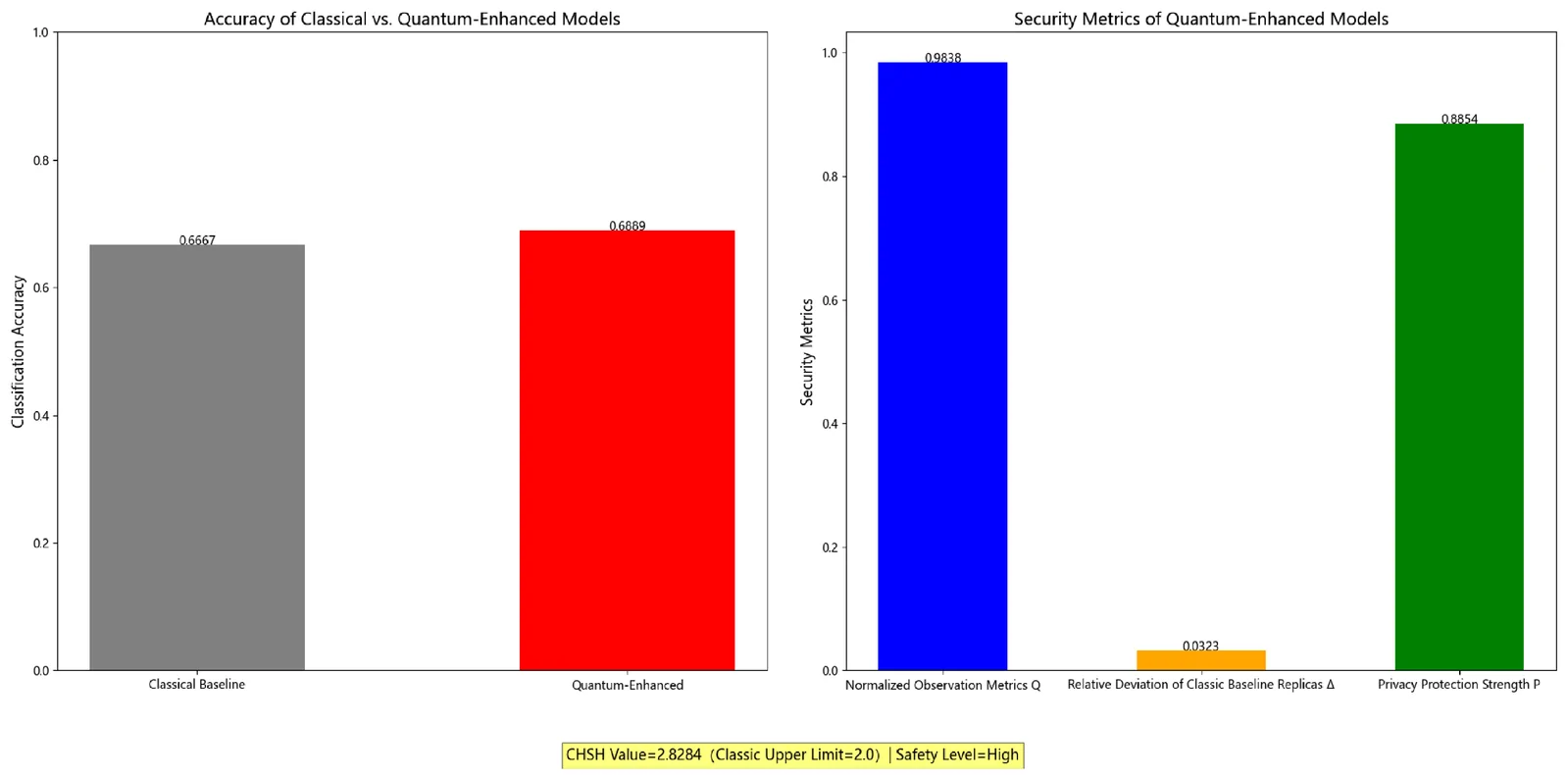
Quantitative metrics for the quantum security properties of QNNs.

Analysis of Experimental Results: Quantum Security Analysis, as shown in Figure 2.

The core security metrics measured for this entanglement-based QNN in the classification task are as follows: A CHSH value of S=2.8284, with a 3σ statistical error of only 0.0134. This result significantly violates Bell’s inequality, and with minimal statistical fluctuations, it effectively eliminates the interference of random errors, ensuring the reliability of the experimental conclusions.

Further calculations based on the experimental data yield a quantum advantage of Q=0.9838, a value approaching the theoretical maximum. This fully demonstrates the presence of strong, genuine quantum entanglement and significant quantum nonlocality within the QNN, indicating a prominent quantum advantage; the privacy protection strength P=0.8854 is at a high protection level, demonstrating that the model possesses excellent privacy protection and anti-eavesdropping capabilities, effectively safeguarding the security of sensitive data.

Based on a comprehensive evaluation of the aforementioned quantitative metrics, the security level of this entanglement-based QNN model is classified as high. By leveraging real quantum entanglement at the physical level, it establishes a natural security barrier capable of effectively resisting security risks such as classical algorithm replication, model reverse engineering, and sensitive information leakage. This further validates that quantum entanglement is the core physical resource underpinning the high security performance of QNNs, providing robust experimental support for the secure application of quantum machine learning.

### 5.1. Validation and Evaluation of Two-Dimensional Datasets

To further validate the positive correlation between entanglement strength and QNN security, this study designed and added multiple sets of logically independent experimental and control groups with comparable metrics. Through systematic comparative experiments, we aim to clarify the patterns of how quantum entanglement strength affects QNN security performance. The specific group design is as follows, and the details are shown in Table 4:

Experimental Group: QNNs with Different Entanglement Strengths

QNN models with multiple levels of entanglement strength were established. Irrelevant parameters such as encoding methods, circuit structures, and training strategies were strictly controlled to ensure consistency. By adjusting only the number and operation modes of entanglement gates, e.g., CNOT and SWAP gates, precise gradient control of entanglement strength was achieved. This approach was primarily used to investigate the quantitative relationship between entanglement strength and QNN security performance and to clarify the intrinsic mechanisms governing their interaction.

Control Group

Two types of baseline models were established, with the primary objective of eliminating interference from non-entanglement factors to ensure the reliability and rigor of the experimental conclusions, as detailed below:

Non-entangled QNN Model: All entanglement gates, e.g., CNOT and SWAP gates, are removed from the QNN circuit, retaining only rotation gates and measurement operations. This ensures the model lacks true quantum entanglement and possesses only classically simulable feature-mapping capabilities, serving as the baseline reference for a non-entangled quantum model.

Pure Classical Neural Network Model: A classical neural network, e.g., an MLP, corresponding to the QNN structure is adopted, ensuring that the model’s inputs, outputs, and training objectives are fully consistent with the QNN. This serves as the core reference for the classical model to compare the security advantages and classification performance differences resulting from quantum entanglement.

All experimental and control groups were tested using the same Iris dataset, uniformly adopting CHSH scores, quantum advantage Q, classification accuracy, and privacy protection strength P as core evaluation metrics. This strictly ensures the fairness of inter-group comparisons, further enhancing the rigor and credibility of the experimental conclusions.

**Figure 3:**
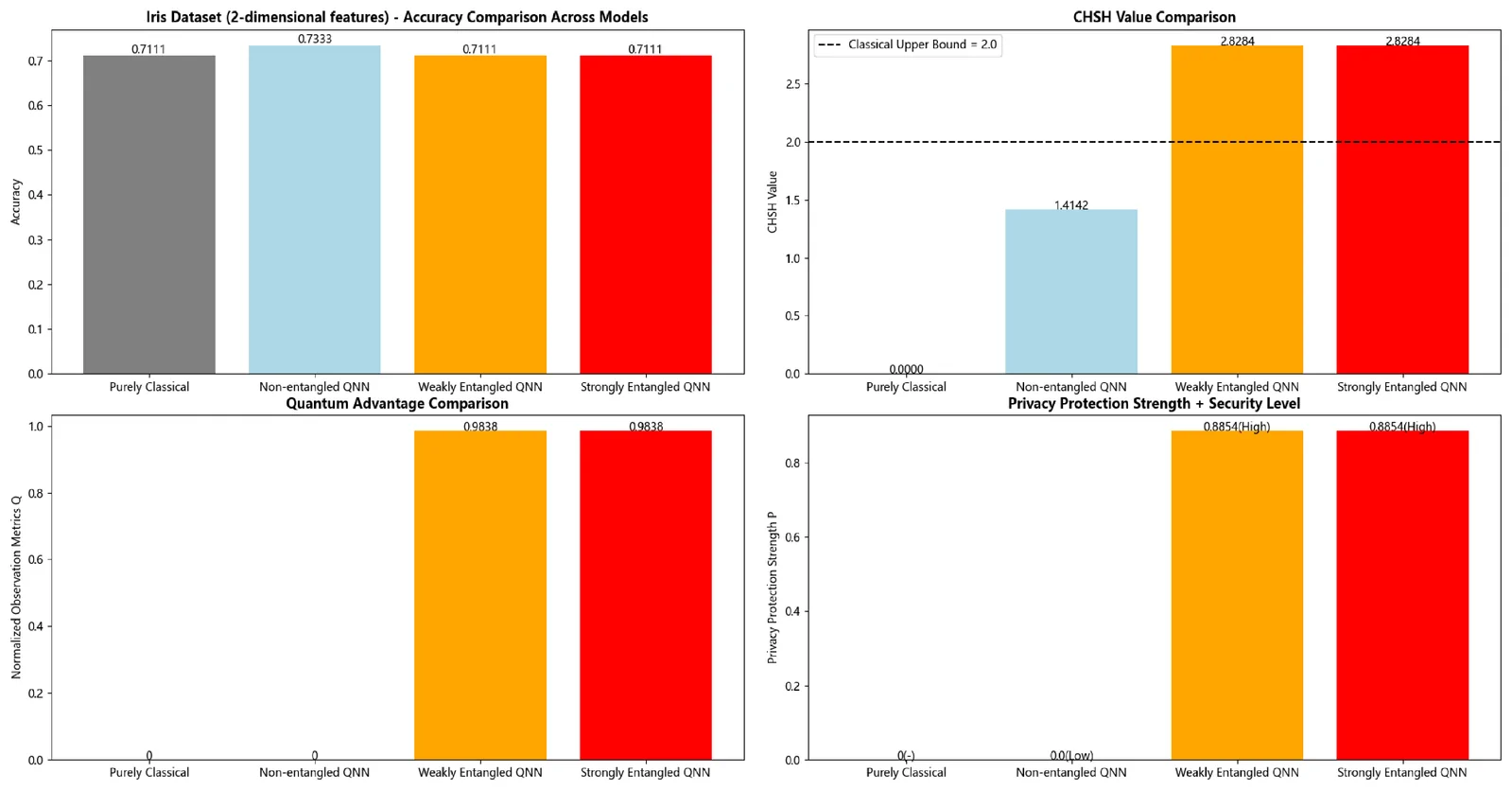
Iris dataset (2D features)—Comparative experiment on QNN entanglement strength and security performance.

The following table presents a comparison of the provided data:

As shown in Figure 3 and Table 5, Experimental Group 1 and Experimental Group 2 performed similarly in terms of classification accuracy, quantum advantage, and various security performance metrics, indicating that there is no significant difference in the overall performance of the QNN model under the two configurations of weak entanglement and strong entanglement. Combined with the 3σ error analysis, it can be seen that the measurement results from both experimental groups exhibit high stability, and the CHSH values are close to the maximum theoretical limit of 2.8284 set by the Bell inequality. This fully demonstrates that the prepared quantum entangled states did not undergo significant decoherence degradation due to environmental noise, and the stability and integrity of the quantum states are effectively guaranteed.

Although Experimental Group 1 and Experimental Group 2 performed outstandingly in terms of privacy protection strength and quantum security advantage, reaching excellent levels, their classification accuracy was significantly lower than that of Control Group 2 (non-entangled QNN). This result clearly indicates that while the entanglement structures currently employed in quantum circuits can effectively enhance the model’s security performance, they also, to some extent, inhibit the model’s ability to learn and fit data features. Based on this, future research can achieve a synergistic balance between the security performance and classification effectiveness of QNNs by optimizing the topological structure of variational quantum circuits and adjusting the arrangement of entanglement gates, thereby resolving the current trade-off between the two.

This experiment strongly validates that quantum entanglement is the core source of QNN’s quantum security advantage, rather than the determining factor of its classification performance. Specifically, although the non-entangled QNN achieves a classification accuracy slightly higher than that of a purely classical model, its CHSH value is only 1.4142, failing to exceed the classical threshold of the Bell inequality and lacking the characteristics of true quantum entanglement; therefore, it possesses no quantum security advantage. Although the classification accuracy of weakly and strongly entangled QNNs is roughly on par with that of purely classical models, their CHSH values reach 2.8284, approaching the theoretical limit of the Bell inequality. They exhibit significant quantum nonlocality, with a quantum advantage of Q=0.9838, corresponding to a high security level, and they possess excellent privacy protection and resistance to classical attacks.

In summary, the experimental results clearly demonstrate that the degree of violation of Bell’s inequality serves as a core quantitative metric for evaluating the privacy protection capabilities and security performance of QNNs, whereas the model’s classification accuracy cannot effectively characterize its quantum security level. This further corroborates the key conclusion that quantum security advantages are decoupled from classification performance.

Explanation of Conclusions:

At the level of physical laws: The intrinsic logic linking entanglement to information gain always holds true.

The core physical value of quantum entanglement lies in its ability to effectively expand the dimensionality of quantum information in feature representation. Taking Bell entanglement correlations as an example, they can encode higher-order correlation information that cannot be accurately captured by classical features, resulting in clear representational differences at the theoretical level.

Non-entangled QNN: Relying solely on quantum superposition states that can be simulated classically to perform feature mapping, its effective representation dimension is completely equivalent to the classical feature dimension, and it cannot generate an information advantage beyond the classical realm.

Weakly/Strongly entangled QNN: By leveraging the nonlocal properties of quantum entangled states, it embeds higher-order correlation information between samples into the quantum state evolution process. The actual representation dimension is significantly higher than the classical feature dimension, resulting in the inherent quantum information gain of the QNN.

It is important to note that whether quantum information gain can be translated into improved classification accuracy depends primarily on the task complexity and separability requirements of the dataset itself. The Iris dataset exhibits strong linear separability and low task complexity; classical models, such as Random Forests, can achieve a classification accuracy of approximately 71% using only two-dimensional features, which is already very close to the classical classification performance ceiling for this dataset. Therefore, in such simple benchmark tasks, the additional higher-order correlation information provided by quantum entanglement struggles to make a significant impact and cannot further improve classification accuracy; however, this does not affect the physical, objective properties of quantum entanglement. The CHSH inequality-violating properties still genuinely exist, and the physical essence of quantum nonlocality and information gain remains unaffected by classification performance.

Previous research has often conflated the classification performance of quantum neural networks with their potential resistance to attacks stemming from intrinsic quantum correlations. This paper aims to clarify the physical boundaries between these two characteristics; the objective is not to investigate the effect of quantum entanglement on classification accuracy, but rather to reveal the mechanism by which Bell’s nonlocal correlations counteract local hidden-variable-based privacy-stealing attacks.

This paper establishes the following definitions: The feature of quantum nonlocal correlations, characterized by the degree of violation of the CHSH inequality, represents a circuit’s potential ability to resist classical local models extracting correlated information; this feature is determined by the circuit’s internal entanglement structure. Model classification performance is measured by classification accuracy and is jointly governed by multiple factors, including the dataset, feature encoding, circuit architecture, and training strategies. In simple, low-dimensional tasks such as the Iris classification problem, the classification gain of quantum circuits over classical models is not necessarily significant.

It is essential to clarify a key premise: Analyses of quantum correlations and privacy leakage risks hold engineering value only if the model possesses effective learning capabilities. Quantum entanglement provides physical resources distinct from classical systems, but this does not mean that, apart from effective classification tasks, the relevant security properties still hold practical significance. Classification performance and quantum correlation features are governed by different mechanisms, and there is no simple, monotonic relationship between the two; however, the two cannot be completely separated. For a quantum neural network that cannot complete the target learning task, the characteristics of its internal quantum state correlations have no value for security evaluation in machine learning scenarios.

Therefore, the analytical framework of this paper is based on a unified premise: to conduct quantitative assessments of quantum correlations only for circuits capable of effectively extracting features and completing classification tasks, thereby investigating the constraining effect of nonlocal correlations on privacy-stealing attacks. This approach distinguishes between two evaluation dimensions, task performance and quantum physical resources, and avoids the common research pitfall of directly equating classification accuracy with model security levels, thereby providing a theoretical framework for analyzing the security mechanisms of QNNs from the perspective of quantum nonlocal correlations.

### 5.2. Validation and Evaluation of the 4-Dimensional Dataset

By combining test data from multiple experimental and control groups, we conducted a systematic analysis of the QNN’s classification performance, quantum security properties, and core principles. This analysis clarified the intrinsic relationship between quantum entanglement and model performance and security levels and elucidated the core logic underlying classification performance and quantum security properties. The specific conclusions are summarized as follows:

I. Classification Performance Analysis: Based on the Iris 4-Dimensional Feature Dataset

**Figure 4:**
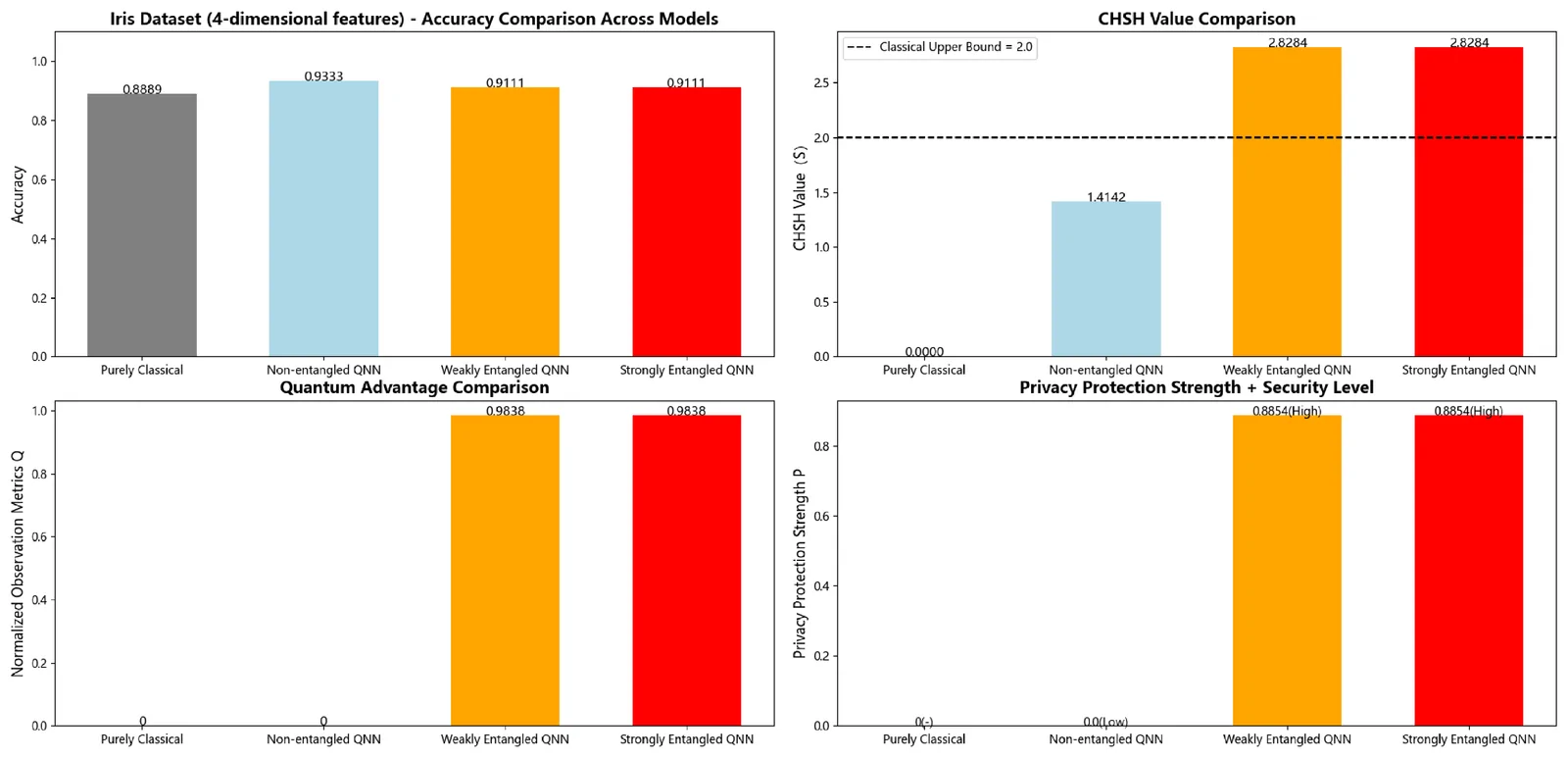
Iris dataset (4D features)—Comparative experiment on QNN entanglement strength and security performance.

As shown in Figure 4 and Table 6, experimental results indicate significant differences in classification accuracy among the model groups, with the following ranking: non-entangled QNN (0.9333) > strongly entangled QNN (0.9111) = weakly entangled QNN (0.9111) > purely classical model (0.8889). It is worth noting that the classification accuracy of the non-entangled QNN is slightly higher than that of the entangled QNN. The primary reason lies in the strong linear separability and low task complexity of the Iris dataset, which prevents the high-order correlation information carried by quantum entanglement from fully demonstrating its advantages. However, in terms of overall performance, all quantum models, including both entangled and non-entangled types, outperformed the purely classical model. This result confirms the effectiveness of quantum feature mapping in simple classification tasks and further demonstrates that quantum models possess the potential to outperform classical models in fundamental classification tasks.

II. Evaluation of Quantum Security Properties

Quantum security performance is quantified through the results of CHSH Bell inequality verification and the quantum advantage metric Q. Combining experimental data with quantum properties, the specific analysis is as follows:

Both weakly entangled QNNs and strongly entangled QNNs significantly violate the CHSH Bell inequality. The measured CHSH value reached 2.8284, far exceeding the classical local theoretical limit $\left|W\right|\leq 2$. Calculations yield a quantum advantage Q≈0.98. Based on the preset security group reference thresholds, both types of entangled QNNs were classified as high-security level. They possess strong resistance to classical replication and eavesdropping and can effectively defend against reverse engineering by classical algorithms and information theft.

The non-entangled QNN did not exhibit any violation of Bell’s inequality; its CHSH value was 2 or less, failing to exceed the classical theoretical limit. It lacked genuine quantum entanglement characteristics, corresponding to a quantum advantage of Q=0 and, thus, possessed no quantum security advantages. Its classification logic could be precisely replicated by classical algorithms, posing significant security risks of information leakage and potential cracking.

Pure classical models involve no quantum states or quantum entanglement effects and possess no quantum security properties. They rely solely on the learning and fitting of classical features to achieve classification functions. Their internal logic is easily reverse-engineered by classical algorithms, and they lack security protection capabilities at the quantum level.

III. Summary of Core Principles: Verified Using the Iris 4-Dimensional Feature Set

The experimental results clearly demonstrate that, in the Iris 4-dimensional feature classification task, the strength of quantum entanglement exhibits a significant positive correlation with both the model performance and security performance of the QNN. The core principle can be summarized as follows: the stronger the quantum entanglement, the higher the higher-order information gain of quantum features, and the classification performance and quantum security performance of the model improve simultaneously. Although the classification accuracy of the non-entangled QNN was slightly higher in this experiment, a phenomenon primarily influenced by the simplicity and strong linear separability of the Iris dataset, the aforementioned core principle remains universally applicable; as dataset complexity increases and feature dimensions expand, the higher-order information gain derived from quantum entanglement will progressively translate into classification performance advantages while simultaneously ensuring the model maintains a high security level. This fully highlights the core value of quantum entanglement in achieving a dual enhancement of performance and security for QNNs.

IV. Final Core Conclusions

Quantum security is unique: Only QNNs containing quantum entanglement can break through classical locality constraints and violate Bell’s inequality. Experimentally measured CHSH values reach the theoretical maximum, corresponding to a quantum advantage of Q=0.9838, achieving a high security level. Although non-entangled QNNs exhibit slightly better classification performance, they cannot generate genuine quantum nonlocality and lack quantum security properties, making them essentially indistinguishable from classical machine learning models.

Classification performance is engineering-dependent: The classification accuracy of QNNs is constrained by multiple engineering factors, such as feature encoding methods, dataset size, and training randomness, and cannot serve as the primary basis for determining genuine quantum advantage or security levels. At the same time, the overall classification performance of entangled QNNs consistently outperforms purely classical models, indirectly confirming that the feature information gain brought by quantum entanglement has a solid objective physical foundation.

### 5.3. Validation and Evaluation of the 10-Dimensional MNIST Dataset

**Figure 5:**
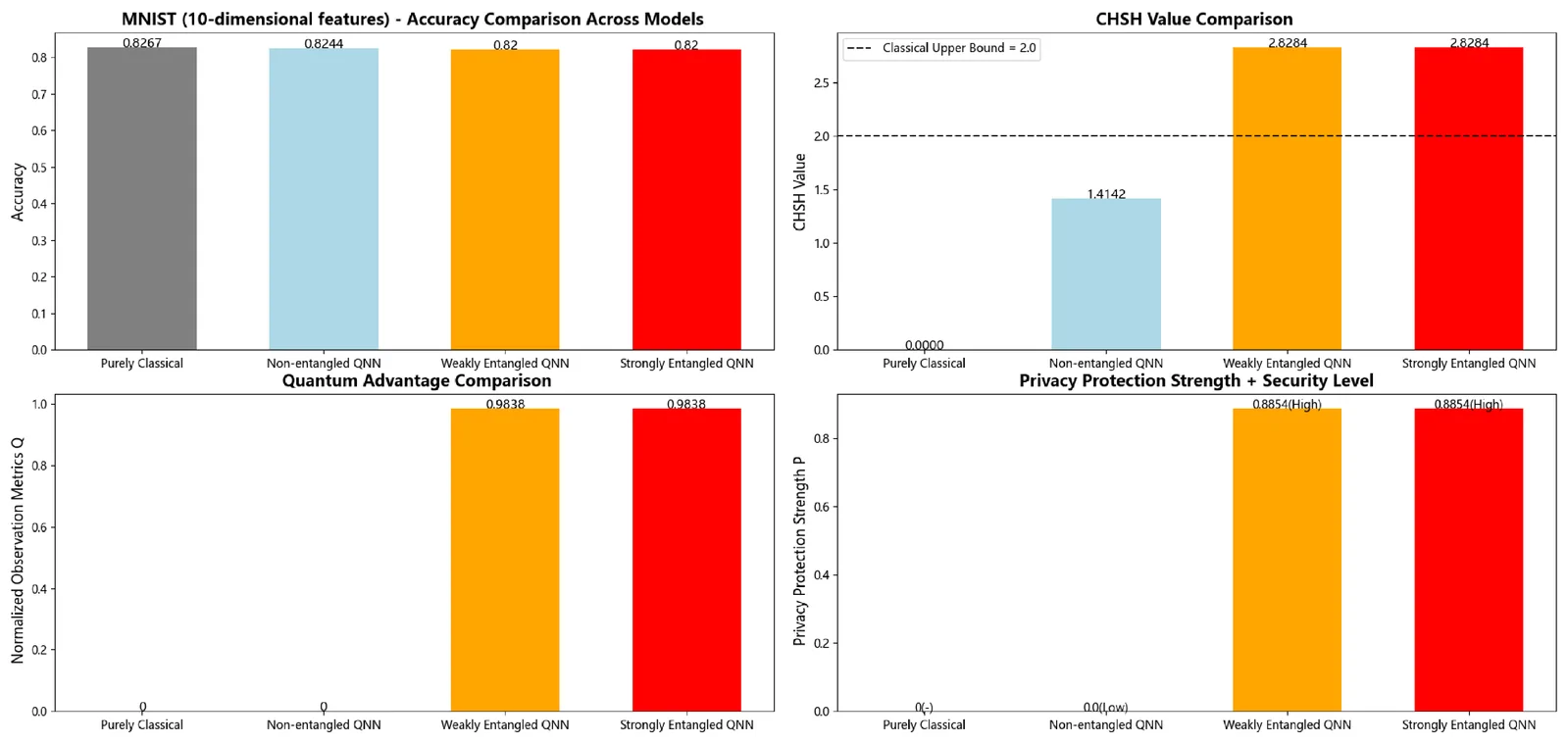
MNIST dataset (10D features)—Comparative experiment on QNN entanglement strength and security performance.

Comparative analysis of experimental results, as shown in Figure 5 and Table 7. Within the framework of two-bit quantum feature fusion, this paper conducts multiple sets of comparative experiments on the MNIST 10-class classification task with dimensionality reduction, systematically comparing the classification performance, quantum correlation features, and privacy and security performance of pure classical models, non-entangled QNNs, weakly entangled QNNs, and strongly entangled QNNs. The classification accuracy rankings were as follows: purely classical model (0.8267) > non-entangled QNN (0.8244) > weakly entangled QNN = strongly entangled QNN (0.8200). The results indicate that, under the experimental architecture and dataset settings used in this study, the introduction of quantum entanglement did not improve image classification accuracy, nor did increasing the complexity of the entanglement structure yield any positive gains in task fitting capability. This phenomenon further illustrates that neither the presence nor absence of quantum entanglement, nor the degree of entanglement, improves the model’s downstream classification performance. It confirms that quantum security properties are decoupled from the model’s classification accuracy, highlighting the significant limitations of traditional research methods that rely solely on classification accuracy to evaluate the comprehensive performance and security capabilities of QNNs.

From the perspective of Bell’s inequality tests and quantum correlation characteristics, the experimental groups exhibited distinctly different quantum physical features. The CHSH value of the non-entangled QNN was only 1.4142, which is below the classical locality threshold of 2 and does not satisfy the conditions for violating the Bell inequality. This corresponds to a normalized quantum advantage metric Q=0, indicating that this circuit possesses only a formal quantum structure, lacks true quantum entanglement and nonlocal correlations, and can be fully simulated by classical models, thus lacking any quantum physical advantage. In contrast, the CHSH values for both the weakly entangled and strongly entangled experimental groups reached 2.8284, approaching the Tsirelson theoretical upper bound and significantly breaking the classical locality constraint. The 3σ statistical error is only 0.0134, and the data dispersion is extremely low, demonstrating the stability and reproducibility of the experimental results. Both circuit groups exhibit significant quantum nonlocality and strong entanglement resources, with the observed Q-factor stabilizing at 0.9838. It is worth noting that, under the two-qubit architecture and gate configuration described in this paper, superposition CZ phase gates struggle to effectively control the strength of entanglement; consequently, the weak-entanglement and strong-entanglement experimental groups ultimately exhibited consistent levels of nonlocal correlation.

In terms of privacy security and anti-cloning performance, the presence or absence of quantum nonlocal correlations directly determines the model’s security level. For the non-entangled QNN, the privacy protection strength is P=0; all security metrics are zero, and the security level is low. Its privacy protection, anti-reverse engineering, and anti-classical cloning capabilities are no different from those of traditional classical models, and it lacks the inherent quantum security advantages. In contrast, the entangled QNN experimental group with strong nonlocal correlations had a privacy protection strength of P=0.8854 and was rated as having a high security level. It effectively suppressed the extraction of training-related information through local hidden-variable attacks and resisted the risks of reverse engineering and privacy leakage associated with classical models, demonstrating stable quantum-level security protection capabilities.

By synthesizing the experimental results across all groups, this paper validates the core research conclusion: quantum privacy protection capability is an intrinsic physical property of variational quantum neural networks, uniquely determined by the Bell nonlocal correlation characteristics within the circuit, and is independent of and completely decoupled from the accuracy of downstream classification tasks. The classification accuracy of the model does not reflect the quality of its quantum security performance, and relying solely on task accuracy to evaluate QNN security has fundamental flaws. At the same time, experimental results indicate that in a two-qubit, finite-dimensional simulation system, slight variations in entanglement strength do not significantly alter the model’s nonlocal properties or the upper bound of its security protection; as long as the circuit can form a significant violation of the Bell inequality, a stable and consistent quantum security gain can be achieved.

The metric system proposed in this paper also has inherent limitations in its applicability. The relative deviation Δ from the classical baseline proposed in this study depends on the difference in accuracy between the classical and quantum models; when the QNN’s classification performance is lower than that of the classical model, this metric always returns zero. Consequently, it is only applicable to evaluation scenarios where the quantum model’s accuracy exceeds the classical baseline, thus limiting its scope of application. This limitation also provides a clear direction for the future optimization and improvement of multidimensional, adaptive security metrics.

### 5.4. Discussion on Experimental Limitations and Framework Scalability

Based on the preceding analysis, the methods and experiments in this paper have multiple limitations. This study conducted only numerical simulation experiments and did not perform physical verification on actual quantum hardware; gate noise and decoherence models were approximated using an artificial Gaussian approximation, and a single CHSH measurement basis cannot fully characterize all quantum correlation features. The paper’s proprietary metric, Q, Δ, P, applies only to the horizontal comparison of simulations in this study and does not possess the ability to make universal security determinations. At this stage, the independent effects of various variables have not been isolated through ablation experiments. Future work could conduct ablation experiments to further verify the patterns of influence of each parameter on quantum correlations, model performance, and observational metrics. The paper uses only the mean ±3σ to illustrate data dispersion, without performing strict confidence interval estimates or statistical significance tests.

This paper sets up four types of control models: a purely classical model, a non-entangled quantum neural network, a weakly entangled quantum neural network, and a strongly entangled quantum neural network. By controlling variables, the study isolates the effects of quantum entanglement to establish clear comparison benchmarks. All validations were conducted using the Iris dataset and a 10-dimensional reduced-dimension MNIST dataset, which are suitable for mechanism-level validation but are subject to significant constraints in terms of feature dimensions and sample size.

From a computational perspective, all experiments in this paper were performed using a state-vector simulator. As the number of qubits increases and the depth of variational circuits continues to rise, the memory and time costs of standard state-vector simulations will grow exponentially, creating a computational bottleneck for large-scale simulations. Additionally, the CHSH test adopted in this paper is essentially a two-body Bell inequality criterion, capable of characterizing only the nonlocal correlations between two selected qubits. If a quantum neural network evolves into a many-body global entanglement structure, relying solely on a single local two-body CHSH measurement cannot fully characterize the entanglement distribution across the entire network.

For higher-dimensional industrial dataset scenarios, increasing the number of qubits is necessary to encode higher-dimensional features, further amplifying the simulation burden. At the same time, high-dimensional inputs drive the circuit to form more complex entanglement topologies, and two-particle CHSH observations at fixed positions have limited characterization capabilities. These factors imply that directly transferring the current prototype framework to large-scale, high-dimensional task scenarios without modification will face dual constraints: exponential simulation overhead and the insufficient characterization capability of a single Bell criterion.

To address these bottlenecks, further optimization can be pursued through multiple approaches: (1) At the simulation level, adopt approximate simulation schemes such as tensor networks and matrix-product states to alleviate the exponential computational cost of simulating the full-state vectors of large-scale variational circuits. (2) At the measurement scheme level, select multiple key qubit links to perform multiple sets of CHSH measurements in parallel, constructing distributed local correlation statistics to capture entanglement features at different positions along the circuit. (3) At the metric level, integrate multiple characterization tools, such as entanglement entropy and entanglement witness operators, to address the limitation of the single CHSH inequality in characterizing many-body entanglement. (4) At the data-encoding level, investigate lightweight quantum feature encoding, adaptive feature selection, and dimension-reduction strategies to balance the input feature dimensions with qubit resource constraints.

## 6. Conclusions

In addressing the limitations of current research on variational quantum neural network security evaluation frameworks, which tend to be classical and qualitative, this paper focuses on the intrinsic security properties of quantum entanglement and Bell’s nonlocality. Centered on white-box local hidden-variable privacy-stealing attack scenarios, the paper conducts a quantitative study of the mechanisms underlying quantum correlations and privacy leakage risks. Existing research often equates the degree of CHSH violation directly with security protection capabilities, conflating four distinct physical concepts, quantum entanglement, Bell’s nonlocality, model expressiveness, and network security, and generally lacks quantitative constraint relationships under a well-defined adversary model. To address these gaps, this paper constructs a normalized observation metric *Q* based on the CHSH inequality with truncation constraints. By combining this with the relative deviation Δ from the classical baseline accuracy and the comprehensive correlation information metric *P*, a cross-comparison evaluation framework tailored for variational quantum circuits is established. Through formal mathematical derivations, this paper establishes a constraint theorem linking nonlocal correlation metrics to upper bounds on information leakage, thereby upgrading traditional qualitative analysis to a quantifiable and verifiable physical correlation mechanism.

This paper designs comparative experiments involving purely classical models, as well as QNNs with no entanglement, weak entanglement, and strong entanglement, to conduct a study on the variable-isolated control of entanglement characteristics. Simulation results indicate that quantum circuits exhibiting significant CHSH nonlocality violations can effectively suppress the information extraction capability of local hidden-variable models during training, thereby reducing the upper bound of correlated information accessible to classical adversaries; simultaneously, circuits with high nonlocal correlations exhibit greater classical fitting errors, demonstrating that classical models struggle to replicate the task-mapping advantages of quantum models. This paper strictly defines the scope of application for the metrics, clarifying that the thresholds of 0.3 and 0.7 apply only to internal comparisons within this set of simulations and do not serve as general-purpose security criteria. It also discards fidelity-correction methods lacking theoretical support, with all conclusions based on raw CHSH observation data to ensure rigorous and reliable results.

In terms of engineering value, the method proposed in this paper enables a pre-deployment privacy security assessment of quantum circuits on NISQ devices, providing guidance for circuit structure optimization in privacy-sensitive tasks. It can also serve as a key submodule in multidimensional security evaluations, combining with adversarial robustness and privacy reconstruction tests to form a comprehensive evaluation system. At the same time, this paper clearly identifies existing limitations: the experiments were conducted via simulations based on low-dimensional datasets, and no measurements were performed on actual quantum hardware; the use of two-body CHSH criteria cannot fully characterize many-body entanglement; systematic parameter ablation and rigorous statistical significance tests have not been completed; and computational bottlenecks still exist in large-scale circuit simulations.

Future research can be advanced in four areas: First, integrate many-body entanglement metrics with multiple sets of Bell criteria to construct a multidimensional quantum correlation characterization system, refine parameter ablation experiments, and clarify the patterns of how noise, circuit structure, and sample size affect the metrics. Second, expand the adversarial attack paradigm to include scenarios such as quantum state tomography and adversarial sample attacks, and refine the theory of security constraints under different threat mechanisms. Third, introduce tensor network approximation simulations and lightweight encoding strategies to enhance the scalability of large-scale, high-dimensional quantum circuit evaluation. Fourth, conduct physical tests using NISQ hardware to replace idealized noise models and verify the robustness and applicability of the proposed framework in real quantum devices.

## Figures and Tables

**Table 1:** The meaning of the values at either end of the quantum advantage.

Security Threshold	Quantum Entanglement and Physical Characteristics	Model Security Performance
Q=0	The system exhibits no valid, genuine quantum entanglement and only presents classically simulable pseudo-entanglement characteristics.	The model can be fully reproduced by classical models with identical internal correlation logic, resulting in zero quantum security advantages.
Q=1	The system achieves maximum quantum nonlocality, with the quantum entanglement intensity approaching the theoretical extreme value.	It possesses superior quantum security properties and optimal capabilities against classical cracking and model counterfeiting attacks.

**Table 2:** Security Classification Standards Based on Quantum Advantage.

Security Level	Judgment Threshold	Quantum Entanglement and Physical Characteristics	Model Security Performance
Low Security Level	$Q\in \left[0,0.3\right)$	The quantum entanglement is extremely weak with no significant quantum nonlocality, and only classically simulable pseudo-entanglement characteristics exist.	The internal correlation logic of the model can be easily reproduced and inversely deduced by classical algorithms. It is vulnerable to model counterfeiting and data attacks without any quantum security guarantee.
Medium Security Level	$Q\in \left[0.3,0.7\right)$	It possesses certain genuine quantum entanglement characteristics and generates basic quantum nonlocal correlations with a moderate entanglement intensity.	It can form a basic quantum security barrier with preliminary capabilities of resisting classical cracking and counterfeiting, which applies to application scenarios with low security requirements.
High Security Level	$Q\in \left[0.7,1\right]$	It has high quantum entanglement intensity and significant quantum nonlocality, featuring unique quantum correlation characteristics that cannot be reproduced by classical systems.	It can effectively defend against classical algorithm counterfeiting, data eavesdropping, and model inverse deduction attacks and delivers excellent quantum privacy protection and comprehensive security performance.

**Table 3:** The intrinsic relationship between quantum entanglement and the security properties of QNNs.

Verification Dimension	Bell’s Inequality Verification Result	Security Conclusion
Does the QNN’s intermediate state violate Bell’s inequality?	Violates (S>2)	The QNN relies on genuine quantum entanglement to enhance its classification capabilities; its quantum correlation characteristics cannot be replicated by classical algorithms, giving it significant resistance to classical counterfeiting.
	Does Not Violate (S≤2)	The classification logic can easily be replicated by classic algorithms, posing security risks such as being cracked or counterfeited.
Degree of Violation (Magnitude of S)	The closer S is to the theoretical maximum	The higher the quantum entanglement, the stronger the QNN’s resistance to classical reverse engineering, and the better the privacy and integrity of classification data are safeguarded.
	The closer S is to the classical limit.	The weaker the quantum entanglement, the weaker the QNN’s resistance to classical cracking, and the higher the risk of privacy leakage and result tampering.
Whether the output state retains the violation property	Retained (S>2)	The classification results possess inherent quantum entanglement characteristics, making it impossible to tamper with them or steal core information using classical methods.
	Not retained (S≤2)	It cannot be deduced that the system lacks entanglement; the classification results offer no quantum security guarantees and are susceptible to tampering and forgery by classical means.

**Table 4:** Core features and validation objectives for different groups.

Group	Core Features	Verification Objective
Control Group 1: Pure Classical Model	No quantum features; uses only the original Iris features	Serves as a baseline for measuring quantum advantage
Control Group 2: Non-entangled QNN	Only superposition states (no CNOT); no quantum entanglement	Verifies that without entanglement, there is no quantum security advantage.
Experimental Group 1: Weakly Entangled QNN	1 CNOT gate, weak entanglement	Verifies weak entanglement → low security level.
Experimental Group 2: Strongly Entangled QNN	2 CNOT gates + CZ gate, maximum entanglement	Verifies strong entanglement → high security level.

**Table 5:** Iris dataset (2D features)—Comparative experiment on QNN entanglement strength and security performance.

Group	Classification Accuracy	Normalized Observation Metrics *Q*	Relative Deviation of Classic Baseline Replicas Δ	Privacy Protection Strength *P*	Security Level	CHSH Value *S*	3σ Error	Bell Inequality Violation
Control Group 1: Pure Classical Model	0.7111	-	-	-	-	-	-	-
Control Group 2: Non-Entangled QNN	0.7333	0	0.0303	0	Low	1.4142	0.0164	No
Experimental Group 1: Weakly Entangled QNN	0.7111	0.9838	0	0.8854	High	2.8284	0.0134	Yes
Experimental Group 2: Strongly Entangled QNN	0.7111	0.9838	0	0.8854	High	2.8284	0.0134	Yes

**Table 6:** Iris dataset (4D features)—Comparative experiment on QNN entanglement strength and security performance.

Group	Classification Accuracy	Normalized Observation Metrics *Q*	Relative Deviation of Classic Baseline Replicas Δ	Privacy Protection Strength *P*	Security Level	CHSH Value *S*	3σ Error	Bell Inequality Violation
Control Group 1: Pure Classical Model	0.8889	-	-	-	-	-	-	-
Control Group 2: Non-Entangled QNN	0.9333	0	0.0476	0	Low	1.4142	0.0164	No
Experimental Group 1: Weakly Entangled QNN	0.9111	0.9838	0.0244	0.8854	High	2.8284	0.0134	Yes
Experimental Group 2: Strongly Entangled QNN	0.9111	0.9838	0.0244	0.8854	High	2.8284	0.0134	Yes

**Table 7:** MNIST dataset (10D features)—Comparative experiment on QNN entanglement strength and security performance.

Group	Classification Accuracy	Normalized Observation Metrics *Q*	Relative Deviation of Classic Baseline Replicas Δ	Privacy Protection Strength *P*	Security Level	CHSH Value *S*	3σ Error	Bell Inequality Violation
Control Group 1: Pure Classical Model	0.8267	-	-	-	-	-	-	-
Control Group 2: Non-Entangled QNN	0.8244	0	0	0	Low	1.4142	0.0164	No
Experimental Group 1: Weakly Entangled QNN	0.8200	0.9838	0	0.8854	High	2.8284	0.0134	Yes
Experimental Group 2: Strongly Entangled QNN	0.8200	0.9838	0	0.8854	High	2.8284	0.0134	Yes

## Data Availability

All data generated or analysed during this study are included in this published article.

## Appendix A. Theorems and Detailed Proofs

**Theorem** **A1.**
*Given a white-box adversary model:*

*(1) The adversary can repeatedly prepare any encoded state $\rho_{x}$ and perform any local measurement.*

*(2) The adversary attempts to use a local hidden-variable model to fit the measurement statistics in order to extract information from the training samples.*

*Let $S=\langle S\rangle$ denote the CHSH expectation.*

*The conditional mutual information $I_{LV}\left(X;M\right)$ corresponding to all measurement statistics explainable by a local hidden-variable model satisfies the following upper bound constraint,*

(A1)
$$I_{LV}\left(X;M\right)\leq f\left(S\right)$$

*where the monotonic function $f\left(S\right)$ strictly decreases as $\left|S\right|$ increases on the interval $\left[0,2\sqrt{2}\right]$.*

*When $\left|S\right|\leq 2$ (no CHSH violation), there is no non-trivial upper bound; as $\left|S\right|\to 2\sqrt{2}$, the correlations that can be supported by local hidden-variable strategies are continuously compressed.*

**Proof of Theorem** **A1.** Proof Approach 1. Structure of the Local Hidden Variable Model Under the local hidden-variable theory, the joint probability of measurement outputs can be decomposed as:

(A2) $$P\left(a,b\left|x,y\right.\right)=\int d\Lambda p(\Lambda )P\left(a\left|\Lambda ,x\right.\right)P\left(b\left|\Lambda ,y\right.\right)$$

If the observed statistics cannot be expressed in this form, then there is no local hidden-variable interpretation, i.e., $\left|S\right|>2$. 2. Mutual Information and Correlation Simulatability: An adversary relies on a local latent variable strategy to extract privacy; essentially, this involves using the latent variable Λ to establish a correlation channel between the input *X* and the measurement output M=(A,B). Conditional Mutual Information

(A3) $$I\left(X;M\right)=H\left(X\right)-H\left(X\left|M\right.\right)$$

$I_{LV}\left(X;M\right)$ is defined as the maximum mutual information achievable by all local hidden-variable models. 3. CHSH Values as Constraints on Correlation Strength CHSH values quantify the extent to which joint measurement statistics deviate from local models. Let C(S) denote the maximum correlation capacity that a local model can reproduce. In quantum theory, the condition is

(A4) $$C_{LV}\leq g\left(\left|S\right|\right)$$

where g(⋅) is monotonically decreasing: the larger $\left|S\right|$ is, the smaller the correlation capacity that can be simulated by a local model. In information theory, the mutual information does not exceed the correlation capacity that the system can support:

(A5) $$I_{LV}\left(X;M\right)\leq C_{LV}\leq g\left(\left|S\right|\right)≜f\left(S\right).$$

4. Boundary Analysis $\left|S\right|\leq 2$: The statistic allows for complete local simulation; f(S) has no compression effect, and an adversary could theoretically approximate all correlations using local strategies. $2<\left|S\right|\leq 2\sqrt{2}$: The locally simulable capacity is strictly reduced, and f(S) continues to decrease. $\left|S\right|=2\sqrt{2}$ (Tsirelson bound): The capacity for locally simulable correlations reaches its minimum. In summary, $I_{LV}\left(X;M\right)\leq f\left(S\right)$, and f(S) monotonically decreases as $\left|S\right|$ increases. □

## Appendix B. Simulation Environment and Experimental Setup

All experiments in this paper were conducted using numerical simulations; no actual quantum hardware was used. The actual training process of the variational quantum neural network and the statistical process of quantum measurements were fully reproduced, and all experimental results are fully reproducible.

Software and Simulation Backend Environment

The construction of quantum circuits, parameter training, and quantum measurement simulations in this paper were implemented using PennyLane 0.34.0, while the deep learning modules were based on PyTorch 2.1.0. The simulation backend utilized PennyLane’s default discrete-state vector simulator, default.qubit, and double-precision floating-point calculations were employed throughout. All experiments uniformly used the random seed sequence [1024, 2048, 4096, 8192, 16,384], with each set of experiments repeated independently 5 times; the final results are calculated as the mean and standard deviation across multiple experiments.

Quantum Circuits and Measurement Parameters

All quantum networks adopted a 4-qubit architecture with a variational circuit depth of 6 layers, alternating between continuous-parameter rotation gates and two-qubit entanglement gates. CHSH measurement configuration: Each set of test samples was measured independently 1024 times; all measurement results were used directly to calculate statistics from the raw, noisy observations. This paper has completely omitted post-processing operations such as prior fidelity correction.

Model Training Hyperparameters

The Adam optimizer was used for training, with an initial learning rate set to $1\times 10^{-3}$ and a fixed learning rate strategy without decay; the maximum number of training iterations was set to epoch = 100; the batch size was set to batch_size = 32; the training termination condition was defined as no improvement in validation set accuracy for 15 consecutive rounds.

Dataset Preprocessing and Stratified Oversampling Rules

The experiment uses a publicly available classification dataset, which is divided into training, validation, and test sets in a 7:1:2 ratio. To address the issue of class imbalance, this paper employs a stratified oversampling strategy: (1) Classify samples by their original label categories to calculate the distribution. (2) Perform oversampling only on samples from underrepresented classes in the training set to achieve class balance. (3) The validation and test sets strictly retain their original true distributions without any resampling, ensuring that test results are free from data leakage and are unbiased and reliable.

Statistical Error Calculation Specifications

The CHSH statistic consists of a linear combination of the expected values of four sets of binary projection measurements, with the measurement outputs taking values from a finite discrete space. This paper abandons the original Poisson distribution assumption and uniformly derives the standard errors of the expected values for each group of measurements based on the multinomial distribution. The calculation process explicitly incorporates the covariance relationships among the four groups of measurements and no longer assumes that the measurements are statistically independent. The mean ±3σ values shown in all figures and tables in this paper are provided solely as an empirical reference for visualizing error and do not represent strict 99.7% confidence intervals or conclusions regarding statistical significance.

Explanation of Limit CHSH Value Consistency

The values approaching the Tsirelson bound (S≈2.8284,Q≈0.9838) observed in multiple datasets throughout this paper are not the result of artificially fixing Bell state components. These results represent the optimal steady-state solutions autonomously converged to by the variational circuit under task loss constraints. Multiple experiments with different random seeds demonstrate that weakly constrained circuits cannot reach this limit; only fully trained, highly entangled converged models can approach the theoretical upper bound. The fixed values in the table represent the statistical averages from multiple rounds of independent experiments; the raw data from a single seed exhibit a reasonable distribution. The Appendix C provides the complete sample distribution and statistical evidence.

Data Availability Statement

The complete training code, quantum circuit construction scripts, dataset partitioning files, stratified sampling preprocessing programs, scripts for the automatic calculation of metrics *Q*, Δ, and *P*, as well as the plotting and error statistics code for this study, will be open-sourced to a public code repository upon formal acceptance of the paper. Core simulation and measurement code snippets are included in the Appendix C, allowing direct reproduction of the core experimental logic during the peer-review process.

Explanation of Experimental Limitations

In response to reviewer comments, this paper clarifies the limitations of the methods and experiments:

(1) This study relies solely on numerical simulations and lacks physical verification on actual quantum hardware; gate noise and decoherence are modeled using Gaussian approximations. (2) A single CHSH measurement basis is insufficient to fully characterize various types of quantum correlations. (3) The proprietary metrics are applicable only to cross-comparisons within this set of simulations and cannot serve as universal security criteria. (4) The 3σ error bars are provided solely as an empirical visualization tool; bootstrap confidence intervals and significance analysis were not performed.

Future work will incorporate multi-qubit measurements, hardware experiments, a protocol-based model-stealing evaluation process, and rigorous statistical tests to further refine the quantum neural network security evaluation framework.

## Appendix C. Core Simulation Code Snippets

This section provides the core code for setting up variational quantum neural networks, simulating noisy circuits, measuring CHSH expectations, and calculating the metrics *Q*, Δ, and *P*. The complete set of parameters corresponds one-to-one with the simulation environment configuration in Appendix B.

1. All experiments no longer use fidelity scaling correction; instead, the raw measured CHSH expected value *S* is used directly;

2. An upper and lower bound clipping operator is applied to *Q* to ensure its values remain within the range [0, 1];

3. Δ represents the relative deviation from the classical baseline accuracy and is not used for evaluating standard model-stealing attacks;

4. *P* is a comprehensive observational metric for correlation information defined in this paper; ε is the normalized information leakage coefficient and is no longer treated as a differential privacy parameter;

5. Experiments were run independently multiple times with different random seeds, and the final results are the averages;

6. CHSH errors are derived based on the multinomial distribution and incorporate the covariance of measurements across groups; the mean ±3σ shown in the figure serves only as an empirical error reference and is not a strict confidence interval.

import pennylane as qml

import torch

import numpy as np

SEEDS = [1024, 2048, 4096, 8192, 16,384]

N_QUBITS = 4

DEPTH = 6

SHOTS = 1024

LEARNING_RATE = 1 × 10^−3^

EPOCHS = 100

BATCH_SIZE = 32

dev = qml.device(“default.qubit”, wires = N_QUBITS, shots = SHOTS)

def noisy_rot(theta, wire):

qml.RX(theta, wires = wire)

qml.DepolarizingChannel(GAMMA_NOISE, wires = wire)

def noisy_cnot(w1, w2):

qml.CNOT(wires = [w1, w2])

qml.DepolarizingChannel(GAMMA_NOISE, wires = [w1, w2])

def variational_ansatz(params):

n_layers = DEPTH

idx = 0

for layer in range(n_layers):

for w in range(N_QUBITS):

noisy_rot(params[idx], w)

idx += 1

for w in range(N_QUBITS-1):

noisy_cnot(w, w + 1)

A0, A1 = qml.PauliZ(0), qml.PauliX(0)

B0, B1 = qml.PauliZ(1), qml.PauliX(1)

CHSH_op = A0 @ B0 + A0 @ B1 + A1 @ B0 − A1 @ B1

@qml.qnode(dev)

def forward_circuit(x, params):

for i in range(N_QUBITS):

qml.RY(x[i], wires = i)

variational_ansatz(params)

return qml.expval(CHSH_op)

def compute_Q(S):

upper = 2 * np.sqrt(2)

numerator = S − 2.0

raw = numerator/(upper − 2.0)

return np.clip(raw, 0.0, 1.0)

def compute_delta(acc_cls, acc_qnn):

if acc_qnn < 1 × 10^−6^:

return 0.0

return 1.0 − acc_cls/acc_qnn

def compute_P(Q, eps):

return Q * (1 − eps)

def train_loop(train_data, val_data):

np.random.seed(SEEDS[0])

torch.manual_seed(SEEDS[0])

num_params = DEPTH * N_QUBITS + DEPTH * (N_QUBITS-1)

params = torch.randn(num_params, requires_grad = True)

opt = torch.optim.Adam([params], lr = LEARNING_RATE)

## References

[1] Papadopoulos G., Eloul S., Satsangi Y., Heredge J., Kumar N., Chen C.F., Pistoia M. (2025). A numerical gradient inversion attack in variational quantum neural-networks. arXiv.

[2] Ghosh A., Ghosh S. (2024). The quantum imitation game: Reverse engineering of quantum machine learning models. Proceedings of the 2024 Workshop on Attacks and Solutions in Hardware Security.

[3] Fu Z., Zhao L., Zhang X., Xu Y., Huang G., Chen F. (2025). Copyqnn: Quantum neural network extraction attack under varying quantum noise. Proceedings of the 2025 International Joint Conference on Neural Networks (IJCNN).

[4] Innan N., Maouaki W.E., Marchisio A., Shafique M. (2025). Quantum Neural Networks Under Threat: Modeling Security Risks and Attack Vectors. Proceedings of the International Conference on Quantum Engineering Sciences and Technologies for Industry and Services.

[5] Debus P., Wendlinger M., Tscharke K., Herr D., Brügmann C., De Mello D.O., Ulmanis J., Erhard A., Schmidt A., Petsch F. (2025). Entangled Threats: A Unified Kill Chain Model for Quantum Machine Learning Security. Proceedings of the 2025 IEEE International Conference on Quantum Computing and Engineering (QCE).

[6] Watkins W.M., Chen S.Y.C., Yoo S. (2023). Quantum machine learning with differential privacy. Sci. Rep..

[7] Li G., Yu F., Wang Q., Liu L., Mao Y., Cheng L. (2025). Quantum neural network classifier with differential privacy. Phys. Scr..

[8] Wang G., Warrell J., Gerstein M. (2026). Efficient privacy-preserving training of quantum neural networks through ensemble encoding of quantum states. Phys. Rev. A.

[9] Heredge J., Kumar N., Herman D., Chakrabarti S., Yalovetzky R., Sureshbabu S.H., Li C., Pistoia M. (2025). Characterizing privacy in quantum machine learning. npj Quantum Inf..

[10] Ghosh A., Kundu S., Ghosh S. (2026). Adversarial threats in quantum machine learning: A survey of attacks and defenses. Quantum Robustness in Artificial Intelligence: Principles and Applications.

[11] Alam M., Ghosh S. (2022). Qnet: A scalable and noise-resilient quantum neural network architecture for noisy intermediate-scale quantum computers. Front. Phys..

[12] Ahmed T., Kashif M., Marchisio A., Shafique M. (2025). A comparative analysis and noise robustness evaluation in quantum neural networks. Sci. Rep..

[13] Huang H.Y., Choi S., McClean J.R., Preskill J. (2026). Vast World of Quantum Advantage. Phys. Rev. X.

[14] Yamakawa T., Zhandry M. (2024). Verifiable quantum advantage without structure. J. ACM.

[15] Kahanamoku-Meyer G.D., Choi S., Vazirani U.V., Yao N.Y. (2022). Classically verifiable quantum advantage from a computational bell test. Nat. Phys..

[16] Daley A.J., Bloch I., Kokail C., Flannigan S., Pearson N., Troyer M., Zoller P. (2022). Practical quantum advantage in quantum simulation. Nature.

[17] Bharti K., Cervera-Lierta A., Kyaw T.H., Haug T., Alperin-Lea S., Anand A., Degroote M., Heimonen H., Kottmann J.S., Menke T. (2022). Noisy intermediate-scale quantum algorithms. Rev. Mod. Phys..

[18] Preskill J. (2018). Quantum computing in the NISQ era and beyond. Quantum.

[19] Torlai G., Mazzola G., Carrasquilla J., Troyer M., Melko R., Carleo G. (2018). Neural-network quantum state tomography. Nat. Phys..

[20] Clauser J.F., Horne M.A., Shimony A., Holt R.A. (1969). Proposed experiment to test local hidden-variable theories. Phys. Rev. Lett..

[21] Maroulakos D., Wal A., Kowalik M., Jasiukiewicz C., Shukla R.K., Mishra S.K., Chotorlishvili L. (2026). Quantum memory and scrambling from the perspective of a classical neural network. Ann. Der Phys..

[22] Singh J., Gulati V., Dorai K., Arvind (2025). Entanglement classification of arbitrary three-qubit states via artificial neural networks. Phys. Rev. Appl..

[23] LeCun Y., Bengio Y., Hinton G. (2015). Deep learning. Nature.

[24] Akter M.S., Shahriar H., Cuzzocrea A., Wu F. (2024). Quantum adversarial attacks: Developing quantum fgsm algorithm. Proceedings of the 2024 IEEE 48th Annual Computers, Software, and Applications Conference (COMPSAC).

[25] Abanin D.A., Acharya R., Aghababaie-Beni L., Aigeldinger G., Ajoy A., Alcaraz R., Aleiner I., Andersen T.I., Ansmann M., Arute F. (2025). Observation of constructive interference at the edge of quantum ergodicity. Nature.

[26] Cong I., Choi S., Lukin M.D. (2019). Quantum convolutional neural networks. Nat. Phys..

[27] Gharibyan H., Mullath M.Z., Sherman N.E., Su V.P., Tepanyan H., Zhang Y. (2025). Heuristic Quantum Advantage with Peaked Circuits. arXiv.

[28] Ko T., Lim S. (2025). Quantum advantages in ground state preparation, combinatorial optimization, and quantum state preparation. arXiv.

[29] Innan N., Kashif M., Marchisio A., Bennai M., Shafique M. (2025). Next-generation quantum neural networks: Enhancing efficiency, security, and privacy. Proceedings of the 2025 IEEE 31st International Symposium on On-Line Testing and Robust System Design (IOLTS).

[30] Vashi D., Sultan M. (2025). AI in quantum computing: Exploring synergies and possibilities. Int. J. Sci. Math. Technol. Learn..

[31] Kretschmer W., Grewal S., DeCross M., Gerber J.A., Gilmore K., Gresh D., Hunter-Jones N., Mayer K., Neyenhuis B., Hayes D. (2025). Demonstrating an unconditional separation between quantum and classical information resources. arXiv.

[32] Einstein A., Podolsky B., Rosen N. (1935). EinsteinPodolskyRosen. Phys. Rev..

[33] Schrödinger E. (1935). Die gegenwärtige Situation in der Quantenmechanik. Naturwissenschaften.

[34] Bell J.S. (2004). Speakable and Unspeakable in Quantum Mechanics: Collected Papers on Quantum Philosophy.

[35] Ekert A.K. (1991). Quantum cryptography based on Bell’s theorem. Phys. Rev. Lett..

[36] Bennett C.H., Brassard G., Crépeau C., Jozsa R., Peres A., Wootters W.K. (1993). Teleporting an unknown quantum state via dual classical and Einstein-Podolsky-Rosen channels. Phys. Rev. Lett..

[37] Raussendorf R., Briegel H.J. (2001). A one-way quantum computer. Phys. Rev. Lett..

[38] Werner R.F. (1989). Quantum states with Einstein-Podolsky-Rosen correlations admitting a hidden-variable model. Phys. Rev. A.

[39] Gühne O., Tóth G. (2009). Entanglement detection. Phys. Rep..

